# The association between physician sex and patient outcomes: a systematic review and meta-analysis

**DOI:** 10.1186/s12913-025-12247-1

**Published:** 2025-01-17

**Authors:** Kiyan Heybati, Ashton Chang, Hodan Mohamud, Raj Satkunasivam, Natalie Coburn, Arghavan Salles, Yusuke Tsugawa, Ryo Ikesu, Natsumi Saka, Allan S. Detsky, Dennis T. Ko, Heather Ross, Mamas A. Mamas, Angela Jerath, Christopher J. D. Wallis

**Affiliations:** 1 Mayo Clinic Alix School of Medicine, Mayo Clinic, Rochester, MN USA; 2 Department of Anesthesia, Sunnybrook Health Sciences Center, Toronto, ON Canada; 3 Temerty Faculty of Medicine, University of Toronto, Toronto, ON Canada; 4 Department of Urology, Houston Methodist Hospital, Houston, TX USA; 5 Center for Outcomes Research, Houston Methodist Hospital, Houston, TX USA; 6 Department of Health Policy and Management, School of Public Health, Texas A&M University, College Station, TX USA; 7 Department of Surgery, Sunnybrook Health Sciences Center, Toronto, ON Canada; 8 Department of Medicine, Stanford University School of Medicine, Palo Alto, CA USA; 9 Division of General Internal Medicine and Health Services Research, David Geffen School of Medicine at UCLA, Los Angeles, CA USA; 10 Department of Health Policy and Management, UCLA Fielding School of Public Health, Los Angeles, CA USA; 11 Department of Epidemiology, UCLA Fielding School of Public Health, Los Angeles, CA USA; 12 Department of Orthopedics, Teikyo University School of Medicine, Itabashi, Tokyo, Japan; 13 Scientific Research WorkS Peer Support Group (SRWS-PSG), Chuo, Osaka Japan; 14 Department of Medicine, Mount Sinai Hospital and University Health Network, Toronto, ON Canada; 15 Institute for Health Policy, Management and Evaluation, University of Toronto, Toronto, ON Canada; 16 Department of Medicine, University of Toronto, Toronto, ON Canada; 17 ICES, 2075 Bayview Avenue, Toronto, ON Canada; 18 Schulich Heart Centre, Sunnybrook Research Institute, Sunnybrook Health Sciences Center, 2075 Bayview Ave, Toronto, ON M4N 3M5 Canada; 19 Division of Cardiology, Peter Munk Cardiac Centre, University Health Network, Toronto, ON Canada; 20 Keele Cardiovascular Research Group, Keele University, Keele, Staffordshire UK; 21 Division of Urology, Department of Surgery, University of Toronto, Toronto, ON Canada; 22 Division of Urology, Department of Surgery, Mount Sinai Hospital, Toronto, ON Canada

**Keywords:** Diversity, Equity, Patient outcomes, Physician sex, Meta-analysis

## Abstract

**Background:**

Some prior studies have found that patients treated by female physicians may experience better outcomes, as well as lower healthcare costs than those treated by male physicians. Physician–patient sex concordance may also contribute to better patient outcomes. However, other studies have not identified a significant difference. There is a paucity of pooled evidence examining the association of physician sex with clinical outcomes.

**Methods:**

This random-effects meta-analysis was conducted according to the PRISMA guidelines and prospectively registered on PROSPERO. MEDLINE and EMBASE were searched from inception to October 4th, 2023, and supplemented by a hand-search of relevant studies. Observational studies enrolling adults (≥ 18 years of age) and assessing the effect of physician sex across surgical and medical specialties were included. The risk of bias was assessed using ROBINS-I. A priori subgroup analysis was conducted based on patient type (surgical versus medical). All-cause mortality was the primary outcome. Secondary outcomes included complications, hospital readmission, and length of stay.

**Results:**

Across 35 (*n* = 13,404,840) observational studies, 20 (*n* = 8,915,504) assessed the effect of surgeon sex while the remaining 15 (*n* = 4,489,336) focused on physician sex in medical/anesthesia care. Fifteen studies were rated as having a moderate risk of bias, with 15 as severe, and 5 as critical. Mortality was significantly lower among patients of female versus male physicians (OR 0.95; 95% CI: 0.93 to 0.97; P_Q_ = 0.13; I^2^ = 26%), which remained consistent among surgeon and non-surgeon physicians (P_interaction_ = 0.60). No significant evidence of publication bias was detected (P_Egger_ = 0.08). There was significantly lower hospital readmission among patients receiving medical/anesthesia care from female physicians (OR 0.97; 95% CI: 0.96 to 0.98). In a qualitative synthesis of 9 studies (*n* = 7,163,775), patient-physician sex concordance was typically associated with better outcomes, especially among female patients of female physicians.

**Conclusions:**

Patients treated by female physicians experienced significantly lower odds of mortality, along with fewer hospital readmissions, versus those with male physicians. Further work is necessary to examine these effects in other care contexts across different countries and understand underlying mechanisms and long-term outcomes to optimize health outcomes for all patients.

**Review registration:**

PROSPERO – CRD42023463577.

**Supplementary Information:**

The online version contains supplementary material available at 10.1186/s12913-025-12247-1.

## Background

A growing body of literature has suggested an association between physician sex and patient outcomes across medical and surgical specialties, including the quality of care, mortality, and length of stay (LOS) [1–4]. A number of studies have demonstrated that female physicians are more likely to follow medical guidelines, offer patient-centred care, provide more preventive care, and exhibit better communication skills [5, 6]. These practices have been postulated to underpin observations that patients treated by female physicians may experience lower odds of mortality and hospital readmission versus male physicians [7, 8]. At the same time, several studies have suggested no significant associations between physician sex and patient outcomes [1, 8–11].

It has also been suggested that patient-physician sex discordance is associated with worse outcomes, especially among female patients [2, 9, 12]. However, there is a more limited consensus on the outcomes of male patients. The underlying mechanisms may include less attention to the severity or different interpretation of symptoms in female patients by male physicians [13], more time spent with patients and better communication skills that could increase rapport [14], lower postoperative pain reporting by patients to male physicians [15, 16], and female patient discomfort for sensitive examinations [2, 17].

To date, there is a paucity of pooled evidence examining the association of physician sex with clinical outcomes in both the medical and surgical setting. Hence, given the mixed findings of prior observational studies, our primary objective was to determine the association between physician sex and outcomes of patients across all medical and surgical specialties by conducting a comprehensive meta-analysis on the topic. Our secondary objective aimed to summarize the relationship between physician–patient sex concordance and patient outcomes.

## Methods

### Eligibility criteria

**Fig. 1:**
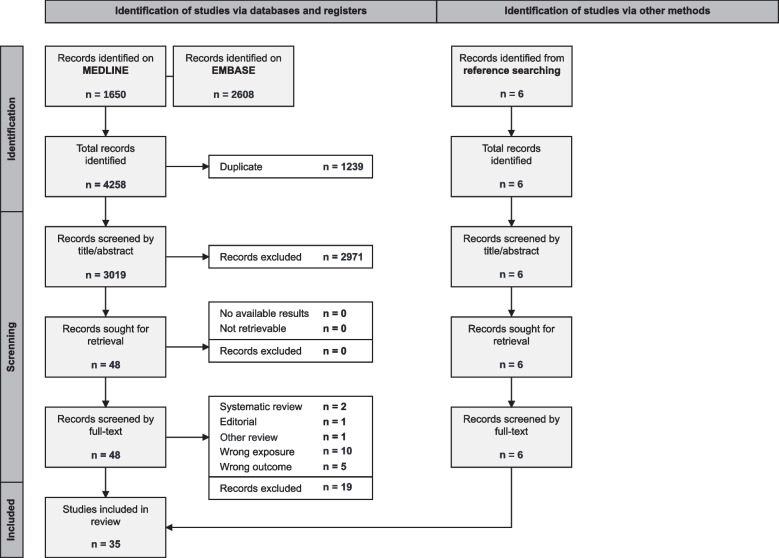
PRISMA flow diagram

This review was prospectively registered on PROSPERO (CRD42023463577) and conducted in accordance with the Preferred Reporting Items for Systematic Reviews and Meta-Analyses framework (PRISMA; Fig. 1, ESM 1 Table 1) [18]. The inclusion criteria consisted of randomized controlled trials (RCTs) or observational studies enrolling adults (≥ 18) investigating the effect of physician sex and/or physician–patient sex concordance on patient outcomes. Patients across the spectrum of medical and surgical care, as well as physicians from all specialties, were included. No relevant RCTs were identified. Non-English language publications were excluded, consistent with prior literature demonstrating limited impact on effect estimates and conclusions [19].

The primary outcome was short-term, all-cause mortality. Studies reporting in-hospital or 30-day mortality were pooled, consistent with the prior literature demonstrating no difference [20, 21]. Secondarily, we examined complications, hospital readmission, and LOS, as defined by individual study investigators.

### Literature search and selection

Searches were conducted by a medical librarian across MEDLINE and EMBASE from inception up to October 4th, 2023. The search strategies are outlined in ESM 1 Tables 2–3. We supplemented this with a hand-search of the bibliographies of relevant studies. The abstract and full-text screening was conducted by KH, AC, and HM through Covidence [22]. Relevant data including study design, author names, year of publication, country of origin, patient type, sample size, and outcomes of interest were extracted using sheets developed a priori. Screening and extraction were conducted independently and in duplicate. All disagreements were resolved through consensus and discussion with senior authors.

### Risk of bias

We assessed the risk of bias (ROB) using the Risk Of Bias In Non-Randomized Studies-of Interventions (ROBINS-I) tool for observational studies [23]. None of the included studies were RCTs and therefore, the use of the Cochrane Risk of Bias Tool for Randomized Trials (RoB-2) was not indicated [24]. Publication bias was assessed using Egger’s test and visualized through contoured funnel plots when more than 10 studies were pooled for a given outcome [25].

### Statistical analysis

We conducted random-effects meta-analyses using the *meta* 4.18 library in R 3.6.3 [26]. Pooled odds ratios (ORs) were calculated with corresponding 95% confidence intervals (CIs). Studies reporting proportions were converted into odds ratios and pooled with other studies reporting odds ratios only. Those reporting odds ratios for male physicians were converted to odds ratios for female physicians, prior to pooling. Studies that could not be pooled and did not have an author response and/or had unavailable data were described narratively. The Mantel–Haenszel and inverse variance methods were used for estimating study weight [27]. Heterogeneity was assessed using Cochran’s Q with a significance threshold of *P*_Q_ < 0.10 as recommended by the Cochrane guidelines [27]. Heterogeneity was further quantified using I^2^ [27]. The I^2^ is a measure of inconsistency between the included studies for a given outcome, with an I^2^ of < 25% interpreted as low, 25 to 75% as moderate, and > 75% as high [27]. The results were visualized using forest plots.

### Subgroup/sensitivity analysis

Pre-planned subgroup analyses were considered based on patient types (surgical versus medical/anesthesia) and on surgical acuity (elective versus emergent) for patients undergoing surgery. However, given the limited number of studies across individual outcomes, we elected to forego subgroup analysis based on surgical acuity. An interaction test was conducted to determine the presence of significant subgroup differences [27]. As Jerath et al. [28] was the only study which focused on anesthesiology, sensitivity analysis was conducted, post hoc, by removing the results from the medical/anesthesia cohort. Bouchgoul et al. [29] and Kobylianskii et al. [30] reported risk ratios, and therefore, we conducted sensitivity analyses by including the unadjusted proportions to derive odds ratios. Chapman et al. [31] reported 30-day patient complication rates based on death or readmission secondary to complications stemming from surgery, and therefore, we conducted a sensitivity analysis excluding these results. Finally, given the inclusion of studies across different continents, we conducted post hoc subgroup analyses based on the geographical location of the study cohorts (North America versus other continents) for the primary outcome of mortality given the limited sample sizes among other outcomes.

### Patient and public involvement

While we recognize the importance of patient and public involvement, this research was an analysis of existing literature, and as such, their involvement was not practical.

## Results

### Screening

We identified 3,019 unique citations from systematic literature searches and 6 from hand searches (Fig. 1). Of these, 54 total full-texts were reviewed, 35 [1, 2, 7–12, 28–54] studies (*n* = 13,404,840) met the inclusion criteria, and data from 30 [1, 7–11, 28–43, 46, 48–54] (*n* = 9,214,223) were included in the meta-analysis.

### Characteristics of included studies

The characteristics of the 35 [1, 2, 7–12, 28–48] included studies (*n* = 13,404,840) are tabulated in Table 1. All studies were observational, with 2 [29, 51] studies (*n* = 22,503) being prospective, 3 [1, 7, 8] studies (*n* = 2,669,667) having a cross-sectional design, and the remainder [2, 9–12, 28, 30–51, 53, 54] (*n* = 10,712,670) being retrospective cohort studies. The majority of studies focused on patients receiving surgical care (20 studies [1–3, 10, 11, 29–36, 40–43, 45, 53, 54]; *n* = 8,915,504), including general, cardiac, orthopedic, obstetric/gynecologic, and ophthalmologic surgery. Of the 15 [7–9, 12, 28, 37–39, 46–48] (*n* = 4,489,336) medical/anesthesia studies, 12 [7–9, 37–39, 46–48, 50–52] focused on general internal medicine or subspecialty internal medicine, 2 [12, 49] focused on emergency medicine and Jerath et al. [28] examined patients undergoing anesthesia for common surgeries. Nineteen [1, 2, 7–9, 11, 12, 28, 30–34, 42, 44, 45, 48, 50, 52] studies included patients receiving care in North America, 9 [29, 36, 38, 43, 46, 47, 51, 53, 54] in Europe, and 7 [10, 35, 37, 39–41, 49] in Asia. Fifteen studies [1, 8, 9, 12, 21, 28–30, 32, 36, 40, 42, 44, 45, 52] were rated as having a moderate ROB, 15 studies [7, 10, 11, 31, 33–35, 37, 39, 41, 43, 46, 47, 49, 51] were rated as severe, and 5 [38, 48, 50, 53, 54] as critical (ESM 1 Table 4). The main threat to internal validity was the risk of confounding.

**Table 1:** Study and patient characteristics

Study Identifier	Year	Country	Study Design	Data Source, Years	Patient Type	Sample Size—Patients		Sample Size—Physicians		Outcomes of Interest
						**Male**	**Female**	**Male**	**Female**	
Surgical										
Blohm, Sandblom, Enochsson, et al.	2023	Sweden	Retrospective (Adjusted)	GallRiks, 2006–2019	Cholecystectomy	51,301^a^	99,175^a^	1,704	849	Mortality (30-day), complications (total), length of stay (prolonged > 3 days)
Bouchgoul, Deneux-Tharaux, Georget, et al.	2023	France	Prospective	TRAAP2 trial data, 2018–2020	Cesarean section	0	4,244^b^	-	-	Complications (post-partum hemorrhage)
Chai, Chen, Lin & Lin	2010	Taiwan	Retrospective (Adjusted)	Taiwan NHIRD, 2004	Cardiac surgery (bypass grafting)	2801	965	149	75	Mortality (in-hospital)
Chapman, Zmistowski, Votta, et al.	2020	United States	Retrospective (Adjusted)	Medicare database	Orthopedic surgery (total knee/hip arthroplasty)	1,518,419		8,778	187	Complications (any postoperative complications)^*^
Etherington, Boet, Chen, et al.	2023	Canada	Retrospective (Adjusted)	Administrative databases, 2007–2017	Non-cardiac surgery	224,896	316,313	S: 1,646 A: 1,077	S: 334 A: 490	Mortality (1-year)
Flodin J, Juthberg R, Edman G & Ackermann	2019	Sweden	Retrospective	Single-center	Orthopedic (Achilles tendon rupture)	89	22	32	9	Complications (perioperative)
Ho, Kuo, Tsai, et al.	2010	Taiwan	Retrospective (Adjusted)	Taiwan NHIRD, 2002–2004	Ophthamologic surgery (scleral buckling or pars plana vitrectomy)	4,514	2,913	-	-	Readmission (180 days)
Jolback, Rogmark, Bedeschi, et al.	2022	Sweden	Retrospective	SHAR, 2008–2016	Hip arthroplasty	5,045	6,948	165	35	Mortality^**^, readmission^**^
Kobylianskii, Murji, Matelski, et al.	2023	Canada	Retrospective (Matched, adjusted)	Multi-center (6 sites), 2016–2019	Hysterectomy	0	2663^c^	23	54	Complications (grade ≥ 2 Clavien-Dindo classification, 30-day)
Mazilescu LI, Bernheim I, Treckmann J, et al.	2023	Germany	Retrospective	Single-center, 2013–2018	Liver transplant recipients	286	139	-	-	Complications (grade ≥ 3 Clavien-Dindo classification)
O’Neill, Lanska & Hartz	2000	United States	Retrospective	PHC4, 1994–1995	Carotid endarterectomy	7,375^d^	5,296^d^	489	18	Mortality (in-hospital)
Okoshi, Endo & Nomura	2022	Japan	Retrospective (Adjusted)	Japanese National Clinical Database, 2013–2017	Gastrointestinal surgery	294,203		9,433	788	Mortality (30-day)
Sharoky, Sellers, Keele, et al.	2018	United States	Retrospective (Matched)	AMA Physician Masterfile, 2012–2013	General surgery	18,632	27,206	152	152	Mortality (in-hospital), complications (any major complications), length of stay
Sun, Boet, Chan, et al.	2021	Canada	Retrospective (Adjusted)	Administrative databases, 2008–2018	Cardiac surgery (bypass grafting and/or valvular)	61,174	18,688	87	11	Mortality (30-day), length of stay
Tsugawa, Jena, Orav, et al.	2018	United States	Cross-sectional (Adjusted)	Medicare inpatient files, 2011–2014	General surgery (≥ 65 years old)	840,402	51,875	41,192	4,634	Mortality (in-hospital or 30-day)
Wallis, Jerath, Coburn, et al.	2021	Canada	Retrospective (Adjusted)	Administrative databases, 2007–2019	Common multispecialty surgeries	559,903	760,205	2,937		Mortality (30-day), complications (any major surgical complications, 30 day), readmission (30-day), length of stay
Wallis, Jerath, Kaneshwaran, et al.	2022	Canada	Retrospective (Adjusted)	Administrative databases, 2007–2019	Common multispecialty surgeries	1,165,711		S: 3,006 A: 1,477		Mortality (30-day), complications (any major surgical complications, 30 day), readmission (30-day)
Wallis, Jerath, Satkunasivam, et al.	2023	United States	Retrospective (Adjusted)	Medicare inpatient files, 2016–2019	Common multispecialty surgeries (≥ 65 years old)	2,732,565		52,852		Mortality (in-hospital or 30-day)
Wallis, Ravi, Coburn, et al	2017	Canada	Retrospective (Matched, adjusted)	Administrative databases, 2007–2015	Common multispecialty surgeries	52,315	52,315	2,540	774	Mortality (30-day), complications (any major surgical complications, 30 day), readmission (30-day), length of stay
Wu, Wu & Weng	2015	Taiwan	Retrospective (Adjusted)	Taiwan NHIRD, 1997–2010	Gynecologic surgery (hysterectomy or hysteropexy)	0	36,909	-	-	Complications (repeat surgery)
Medical + Anesthesia										
Becker, Siry-Bove, Shelton, et al.	2022	United States	Retrospective	Single-center (CCAR), 2013–2018	Post-cardiac arrest	217	123	-	-	Mortality (30-day)
Berg, Hurtig & Steinsbekk	2022	Norway	Retrospective (Adjusted)	Single-center, 2005–2017	General internal medicine	5,682^f^	5,377	33	8	Mortality (in-hospital), readmission (30-day), length of stay
Dwyer & Kalın	2021	Sweden	Retrospective	Single-center, 2008–2010	General internal medicine	422	404	-	-	Mortality (30-day), Readmission (14-day)
Dziewierz, Vogel, Zdzierak, et al.	2023	Poland	Retrospective (Adjusted)	ORPKI database, 2014–2020	Coronary angiography and percutaneous coronary intervention	393,464	188,280	782	34	Mortality (in-hospital), complications (periprocedural)
Greenwood, Carnaha & Huang	2018	United States	Retrospective (Matched)	Administrative databases, 1991–2010	Emergency medicine (myocardial infarction)	338,642^ g^	243,203^ g^	-	-	Mortality (in-hospital), length of stay
Haubitz-Eshchelbach, Mirsada, Sebastian, et al.	2019	Switzerland	Prospective (Adjusted)	Single-center, 2013–2016	General internal medicine	10,058	8,201	62^ h^	32^ h^	Mortality (in-hospital)
Jerath, Satkunasivam, Kaneshwaran, et al.	2023	Canada	Retrospective (Adjusted)	Administrative databases, 2007–2019	Anesthesia for any common surgery	1,104,657	151,054	1,012	465	Mortality (30-day), complications (any major complications, 30-day), readmission (30-day), length of stay
Nakayama, Morita, Fujiwara & Komuro	2019	Japan	Retrospective	Single-center, 2012–2018	Internal medicine (inpatient cardiology)	7,550	1,994	68	20	Mortality (in-hospital, < 30 days), readmission
Meier, Yang, Liu, et al.	2019	United States	Retrospective	Inpatient cardiac arrest database from 2 sites, 2005–2017	Internal medicine (inpatient cardiac arrest)	688	394	-	-	Mortality (in-hospital)
Rifkin, Holmboe, Scherer, et al.	2004	United States	Retrospective (Adjusted)	Single-center, 2001	General internal medicine	5,497^i^	5,891^i^	207		Length of stay
Sagy, Fuchs, Mizrakli, et al.	2018	Israel	Retrospective	Single-center, 2011–2012	Emergency medicine (critically ill)	475	356	-	-	Mortality (in-hospital), length of stay
Sergeant, Saha, Shin et al.	2021	Canada	Cross-sectional (Adjusted)	GEMINI study database, 2010–2017	General internal medicine	84,221^j^	87,402^j^	118	54	Mortality (in-hospital), readmission, length of stay
Shen, Li, Wu & Yang	2021	China	Retrospective (Adjusted)	UEBMI database, 2018–2019	General internal medicine	79,805		3,993		Readmission (30-day)
Tsugawa, Jena, Figueroa, et al.	2017	United States	Cross-sectional (Adjusted)	Medicare inpatient files, 2011–2014	General internal medicine (≥ 65 years old)	635,726^ k^	980,129^ k^	39,593	18,751	Mortality (30-day), readmission (30-day)
Yelavarthy, Seth, Pielsticker, et al	2021	United States	Retrospective	BMC2 quality improvement database, 2010–2017	Percutaneous coronary intervention	159,969	79,453	385	18	Mortality (in-hospital), complications (AKI)

*Abbreviations*: *GallRiks* Gallstone Surgery and Endoscopic Retrograde Cholangiopancreatography, *TRAAP2* Tranexamic Acid for the Prevention of Blood Loss after Cesarean Delivery, *NHIRD* National Health Insurance Research Database, *S* Surgeons, *A* Anesthesiologists, *PHC4* Pennsylvania Health Care Cost Containment Council, *AMA* American Medical Association, *SHAR* Swedish Hip Arthroplasty Register, *CCAR* Colorado Cardiac Arrest Registry, *ORPKI* Polish National Registry of PCI, *GEMINI* General Medicine Inpatient Initiative, *UEBMI* Urban Employee Basic Medical Insurance, *BMC2* Blue Cross Blue Shield of Michigan Cardiovascular Consortium, *AKI* Acute kidney injury

^*^30-day patient complication rates (based on death or readmission secondary to complications stemming from the surgery) were determined separately for THA and TKA for each surgeon. A readmission was deemed a complication if the primary diagnosis for readmission was considered to be related to the index procedure. Relatedness was determined by a panel of physicians and surgeons assessing each identified readmission diagnostic code. Breakdown of 30-day death or readmission not available

^**^Unpublished data

^a^Total sample size – 150,509; missing patient sex—33. Analyses based on total outcome-specific sample sizes reported by the study: Mortality—149,703; Total complications—146,416; Length of stay—147,304

^b^Analyses based on total sample size of 4,153

^c^Matched cohort total sample size of 1,926

^d^Total sample size – 12,725

^e^Mortality analysis based on admitting physician sex. Sex concordance data presented for care team

^f^Mortality analysis based on total sample size of 8,657. Length of stay could not be pooled. Based on number of admissions

^g^Matched total sample size – 134,426 (67,213 male and 67,213 female patients)

^h^Data based on attending-level physicians

^i^Analyses based on 11,383 patients; missing complete data – 5

^j^Total sample size – 171,625; missing patient sex – 2

^k^Analyses based on total outcome-specific sample sizes reported by the study: Mortality – 1,283,621; Readmissions – 1,249,210

### All-cause mortality

**Fig. 2:**
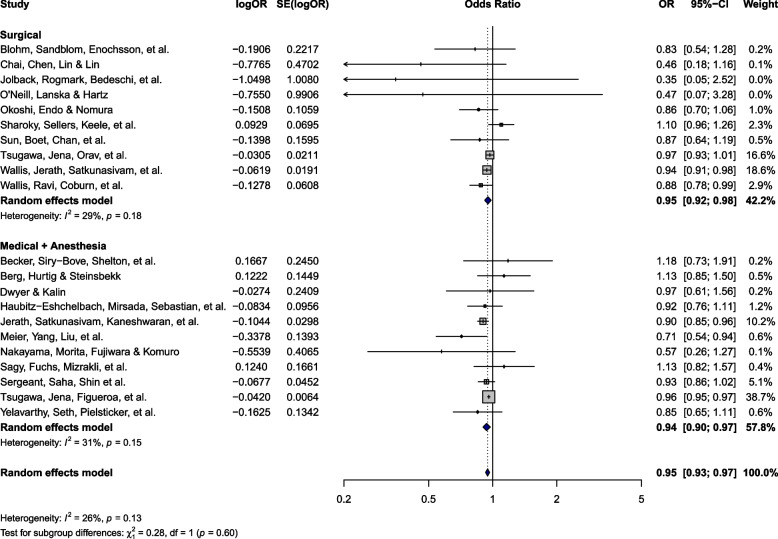
Mortality forest plot. Abbreviations: OR – Odds ratio; SE – Standard error; CI – Confidence interval

Twenty-one [1, 7–11, 28, 32–38, 42, 43, 46, 48–51] studies (*n* = 7,217,390) reported in-hospital or 30-day mortality, of which 10 [1, 10, 11, 32–36, 42, 43] focused on surgical care and 11 [7–9, 28, 37, 46, 49–51] examined patients receiving medical/anesthesia care (Fig. 2). Eleven studies were rated as having a severe ROB, 7 rated as moderate, and 3 as critical (ESM 1 Table 4). Patients treated by female versus male physicians had significantly lower mortality (OR 0.95; 95% CI: 0.93 to 0.97). There was non-significant, low to moderate heterogeneity across studies (P_Q_ = 0.13; I^2^ = 26%). Egger’s test did not reveal significant publication bias (P_Egger_ = 0.08; ESM 1 Fig. 1).

**Fig. 3:**
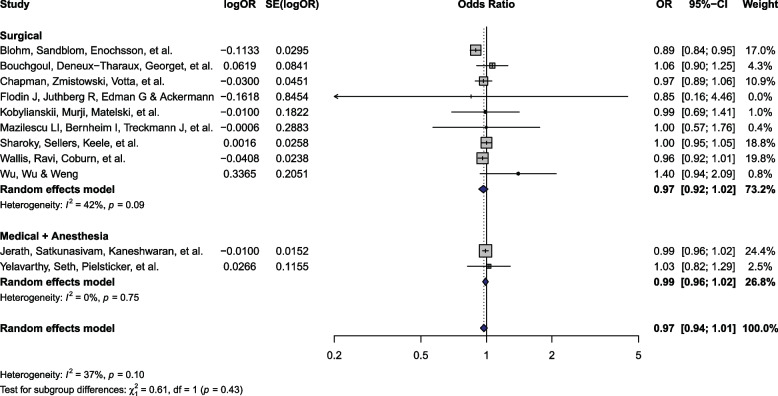
Complications forest plot. Abbreviations: OR – Odds ratio; SE – Standard error; CI – Confidence interval

There were no significant subgroup interactions between care settings (surgical versus medical/anesthesia) (P_interaction_ = 0.60; Fig. 2). There was a similar magnitude of effect among the surgical (OR 0.95; 95% CI: 0.92 to 0.98) and medical/anesthesia (OR 0.94; 95% CI: 0.90 to 0.97) subgroups. The results remained consistent following the sensitivity analysis (ESM 1 Fig. 2). This effect remained significant across the 12 studies (*N* = 6,719,608) which included data from North America (OR 0.95; 95% CI: 0.93 to 0.97; ESM 1 Fig. 3). While there were no significant subgroup interactions (P_interaction_ = 0.72), the effect size was no longer significant among the 9 studies (*N* = 497,782) from other continents (OR 0.93; 95% CI: 0.83 to 1.03; ESM 1 Fig. 3). The data from individual studies are summarized in ESM 2.

### Complications

**Fig. 4:**
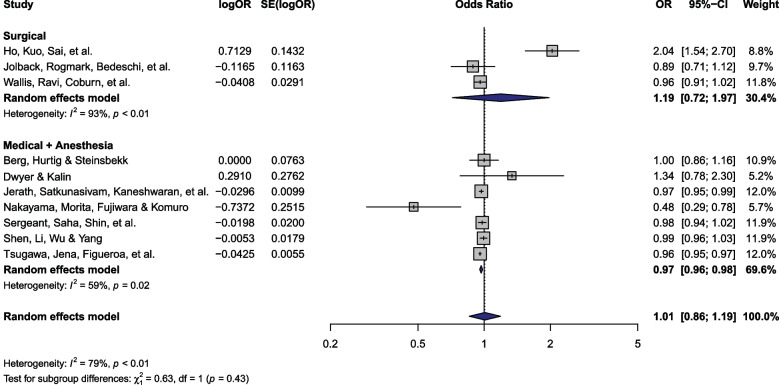
Hospital readmission forest plot. Abbreviations: OR – Odds ratio; SE – Standard error; CI – Confidence interval

Eleven [9, 11, 28–32, 41, 43, 53, 54] studies (*n* = 3,264,051) reported complications, of which 9 [11, 29–32, 41, 43] enrolled patients undergoing surgery and 2 [9, 28] included those receiving medical/anesthesia care (Fig. 3). Six studies were rated as having severe ROB, with the other 5 rated as moderate. There was no significant difference in complications among patients of female versus male physicians (OR 0.97; 95% CI: 0.94 to 1.01). There was non-significant, moderate heterogeneity across studies (P_Q_ = 0.10; I^2^ = 37%). There was no evidence of publication bias (P_Egger_ = 0.62; ESM 1 Fig. 4).

There were no significant subgroup differences between care settings (P_interaction_ = 0.43; Fig. 3). The findings remained non-significant across both subgroups and following sensitivity analyses (ESM 1 Figs. 5–7). The data from individual studies are summarized in ESM 2.

### Hospital readmission

Ten [7, 8, 28, 32, 37–40, 46] studies (*n* = 2,811,110) reported hospital readmission, of which 3 [32, 36, 40] included surgical patients and 7 [7, 8, 28, 37–39, 46] enrolled those receiving medical/anesthesia care (Fig. 4). Five studies were rated as having moderate, 4 as severe, and 1 as critical ROB (ESM 1 Table 4). There was no significant difference in readmission among patients of female versus male physicians (OR 1.01; 95% CI: 0.86 to 1.19). There was significant, high heterogeneity across studies (P_Q_ < 0.01; I^2^ = 79%). There was no evidence of publication bias (P_Egger_ = 0.36; ESM 1 Fig. 8).

 Despite non-significant (P_interaction_ = 0.43) subgroup differences, there were significantly fewer hospital readmissions among female physicians when compared with male physicians (OR 0.97; 95% CI: 0.96 to 0.98; Fig. 4) in the medical/anesthesia subgroup. There were no significant differences across the surgical subgroup (Fig. 4) and the findings remained consistent following the sensitivity analysis (ESM 1 Fig. 9). The data from individual studies are summarized in ESM 2.

### Hospital length of stay (LOS)

Seven [8, 11, 28, 32, 33, 43, 52] studies (*n* = 1,726,353) reported hospital LOS, of which 4 [11, 32, 33, 43] enrolled surgical patients and 3 [8, 28, 52] included those receiving medical/anesthesia care (ESM 1 Fig. 10). An additional study by Berg et al. [46] (*n* = 11,059) reported on LOS which could not be pooled. Four studies were rated as having severe ROB, with the remaining 4 rated as moderate (ESM 1 Table 4). Overall, there was no significant difference in hospital LOS among patients of female versus male physicians (OR 0.97; 95% CI: 0.90 to 1.04). There was significant, high heterogeneity across studies (P_Q_ < 0.01; I^2^ = 91%).

There were no significant subgroup differences between care settings (P_interaction_ = 0.36) and the findings remained non-significant among both subgroups (ESM 1 Fig. 10). Following sensitivity analysis by excluding Jerath et al. (*N* = 1,165,711) [28], medical patients (*N* = 183,008) treated by female physicians had significantly higher LOS (OR 1.03; 95% CI: 1.01 to 1.06; ESM 1 Fig. 11). The data from individual studies are summarized in ESM 2.

### Physician–patient sex concordance

Nine [2, 9, 12, 42, 44, 45, 47, 48] studies (*n* = 7,163,775) reported on sex concordance, of which 4 [2, 42, 44, 45] enrolled surgical patients and 5 [9, 12, 47–49] included patients receiving medical/anesthesia care (Table 1). Three [44, 45, 48] studies specifically looked at the effect of care team sex concordance on patient outcomes. Seven studies were rated as having a moderate ROB with 2 rated as severe (ESM 1 Table 4).

Wallis et al. [2] (*n* = 1,320,108) found that, among female patients, sex discordance translated to significantly higher mortality versus sex concordance (aOR 1.32; 95% CI: 1.14 to 1.54) in Canadian health datasets. Conversely, among male patients, sex discordance resulted in lower odds of mortality (aOR 0.87; 95% CI: 0.78 to 0.97). Similarly, another study [42] (*n* = 2,732,565), using American Medicare data, found that patient-surgeon sex concordance was associated with lower mortality for female patients (adjusted risk difference [aRD] −0.15 pp; 95% CI: −0.32 to −0.05 pp), but higher mortality for male patients (aRD 0.24 pp; 95% CI: 0.08 to 0.40 pp). Greenwood et al. [12] (*n* = 581,845) found that female patients treated by male physicians had lower mean survival than male patients treated by male physicians (matched *n* = 119,304; mean [SD] 0.852 [0.355] versus 0.874 [0.332]) across American emergency departments. This discrepancy was less pronounced among male and female patients with female physicians (matched *n* = 15,122; male patient/female physician 0.872 [0.334] versus female patient/female physician 0.869 [0.338]). Conversely, Yelavarthy et al. [9] (*n* = 239,422) and Dziewierz et al. [47] (*n* = 581,744) showed no significant association between proceduralist-patient sex concordance and mortality. Sagy et al. [49] (*N* = 831) also found no significant association between physician–patient sex concordance and in-hospital or longer-term mortality. The data on other outcomes are outlined in ESM 2.

## Discussion

### Main findings

Across the 35 included studies [1, 2, 7–12, 28–48, 53, 54] (*n* = 13,404,840), of which 30 [1, 7–11, 28–43, 46, 48–54] (*n* = 9,214,223) were included in the meta-analysis, there was significantly lower odds of mortality among patients of female versus male physicians (OR 0.95; 95% CI: 0.93 to 0.97), an effect which was consistent across surgical and medical/anesthesia care. Fewer studies evaluated complications, hospital readmission, and LOS. In general, these analyses did not demonstrate significant differences based on physician sex, apart from lower odds of hospital readmission identified among patients receiving medical/anesthesia care from female versus male physicians (OR 0.97; 95% CI: 0.96 to 0.98). A systematic review of 9 [2, 9, 12, 42, 44, 45] studies (*n* = 7,163,775), revealed that physician and patient sex concordance was typically associated with better patient outcomes, especially among females managed by female physicians.

### Findings in context

While this study represents, to the best of our knowledge, one of the first quantitative meta-analysis assessing the association between physician sex and patient outcomes, our findings align with prior observational studies [1, 2, 4, 32]. Specifically, we found significantly lower mortality among patients of female versus male physicians, which remained consistent among patients receiving surgical and medical/anesthesia care. Other studies which also reported similar findings generally had larger sample sizes [1, 2, 32] with smaller studies reporting no difference with wide confidence intervals. Of note, many of these larger studies with positive findings analyzed cohorts from North America. Our analyses revealed that while the significantly lower mortality among patients of females persisted across studies from North America, there was no significant difference when pooling data from other continents, despite no significant subgroup interactions. It is unknown whether there are regional and cultural differences in factors such as patient-physician relationships, expectations, and communication styles that may affect the generalizability of our findings that were largely seen in studies from North America. We are unable to infer any regional differences given the variability in data sources, and fewer number of studies from other continents that may have led to decreased power in our analyses. There were also potentially fewer female physicians represented in the study cohort; however, the full details of unique physician composition were not consistently present. Therefore, larger prospective studies from various countries are required to confirm these findings.

Several underlying mechanisms have been suggested for these findings. Specifically, female physicians have been shown to adhere more closely to clinical guidelines and offer more evidence-based care [5, 6]. Prior publications have also suggested that this difference may be attributable to better communication skills and a greater willingness to work with the interprofessional team when compared to male physicians [55]. Female physicians have also been shown to exhibit more risk-averse decision-making [17, 55], which may influence patient selection for surgery. Some studies have also reported better scores and skill acquisition among female versus male medical students [56, 57]. We found significantly lower hospital readmission among medical/anesthesia patients managed by female physicians which is consistent with prior observational studies [7, 28, 37]. There were no differences among those receiving surgical care; however, only 3 studies [32, 36, 40] reported readmission among this subgroup. We also did not find any significant difference in complications or hospital LOS. There have been fewer observational studies reporting on these outcomes and further study is required to determine whether a significant association exists.

With respect to patient-physician sex concordance, while there is evidence that overall sex discordance is associated with worse outcomes, female patients tend to experience better outcomes with female physicians versus when treated by male physicians. However, the differences in outcomes for male patients are smaller. The underlying mechanisms may include less attention to the severity or different interpretation of symptoms in female patients by male physicians [13], more time spent with patients and better communication skills that could increase rapport [14], lower postoperative pain reporting by patients to male physicians [15, 16], and female patient discomfort for sensitive examinations [2, 17]. There is also limited evidence on the effect of surgeon-anesthesiologist sex concordance on patient outcomes, with one study reporting no significant difference [45] and another noting lower 1-year mortality among those treated by sex-discordant surgeon-anesthesiologist teams [44]. In the latter, they note that sex discordance among teams (i.e. surgeon’s sex differing from most of the team) may result in better teamwork in the OR, regardless of specific sex. Future studies should continue to investigate underlying explanations, including an approach that considers intersectionality. Additionally, future investigations should involve patient perspectives in study design, as well as data interpretation and implications to better understand potential mechanisms and interventions for the associations between physician sex and patient outcomes.

### Strengths & limitations

This systematic review and meta-analysis is among the first, to our knowledge, to quantitatively evaluate the effect of physician sex on the outcomes of adult patients receiving medical or surgical management. In addition, we analyzed medical and surgical studies separately and in combination, as determined a priori, to explain possible sources of heterogeneity and improve the applicability of our findings.

Despite the strength of these data, our systematic review noted several limitations in the published literature. Most of the studies were retrospective with many having a severe risk of bias due to the potential for residual confounding, despite adjustment in select studies. While we attempted to conduct several sensitivity analyses, the relatively large proportion of studies with high risk of bias precluded us from conducting analyses without their inclusion. Many studies also utilized large databases and diagnosis codes to ascertain physician and patient characteristics, introducing a risk of misclassification. Although databases are excellent resources to capture healthcare interactions, they vary in patient cohorts captured, access to different healthcare, and accuracy of codes that could impact data accuracy and generalizability [58, 59]. Additionally, while prior studies have demonstrated no difference when pooling in-hospital and 30-day mortality [20, 21], the included studies also often reported on the other outcomes of interest at different time points. Observational studies tend to overestimate effect sizes demonstrating benefit, as well [60]. Furthermore, there was a limited number of studies across outcomes other than mortality. This may have affected our ability to detect significant associations, as well as subgroup differences between patient types. Specifically, while surgery represents around 29% of overall care, a larger proportion of the pooled studies investigated the outcomes of patients undergoing surgery [61]. This highlights the need for more studies among those receiving non-surgical management. The literature has also often focused on biological sex as a binary variable, with more limited data on gender. In our analyses, we also found significant heterogeneity among studies that was not fully explained by additional analyses. Additionally, only studies published in English were included; however, prior literature suggests no meaningful effect on pooled estimates or conclusions [19]. We conducted extensive systematic and hand searching, and Egger’s test did not reveal any significant publication bias. Studies reporting on physician–patient sex concordance could not be pooled given the limited sample size and heterogeneous reporting.

## Conclusion

Patients treated by female physicians experienced lower mortality, and those receiving medical/anesthesia care from female physicians experienced fewer hospital readmissions versus male physicians. Physician–patient sex concordance was also associated with better patient outcomes, especially among female patients. Further work is necessary to examine these effects in other care contexts across different countries, as well as to understand underlying mechanisms and long-term outcomes to better inform healthcare strategies and patient care.

## Supplementary Information

Supplementary Material 1.

Supplementary Material 2.

## Data Availability

Available upon request from the corresponding authors (AJ and CJDW).
