# Supplementary material for: The association between physician sex and patient outcomes: a systematic review and meta-analysis
Source: BMC Health Serv Res. 2025 Jan 17;25:93. doi: 10.1186/s12913-025-12247-1 (PMC11740500; doi:10.1186/s12913-025-12247-1)
Supplement: Supplementary file 1 — Supplementary Material 1. [file 12913_2025_12247_MOESM1_ESM.docx]

**Electronic Supplementary Materials 1**

**The association between physician sex and patient outcomes: A systematic review and meta-analysis**

Kiyan Heybati BHSc^1^, Ashton Chang MD^2^, Hodan Mohamud BSc^3^, Raj Satkunasivam MD MSc^4,5,6^, Natalie Coburn MD MPH^7^, Arghavan Salles MD PhD^8^, Yusuke Tsugawa MD PhD^9,10^, Ryo Ikesu MD,^11^ Natsumi Saka MD PhD,^12,13^ Allan S. Detsky MD PhD^14,15,16^, Dennis T Ko MD MSc^15,16,17,18^, Heather Ross MD MHSc^19^, Mamas A. Mamas MD DPhil^20^, Angela Jerath MD MSc^2, 15, 17, 18^*****, Christopher JD Wallis MD PhD^21,22^*

**Equal contribution senior authors*

**Table of Contents:**

**Table 1.** Preferred Reporting Items for Systematic Reviews and Meta-Analyses (PRISMA) 2020 Checklist…………………………………………………………………….……………………2

**Table 2.** MEDLINE search strategy…...…………………………………..……………………..7

**Table 3.** EMBASE search strategy..…..………………………………………………………….9

**Table 4.** ROBINS-I risk of bias ratings..…………………….………….…………...……….11-12

**Table 5.** List of excluded studies during full-text screening…………………………………13-14

**Figure 1.** Mortality funnel plot..…..………………………………………………………….….15

**Figure 2.** Forest plot for mortality sensitivity analysis..……..……………...……………….….16

**Figure 3.** Forest plot for mortality subgroup analysis stratified by North America versus

Other Continent location..……..….… ..…. ..…. ..…. ..…. ..…. .... ..…. ..…. ..…. ..…….….….17

**Figure 4.** Complications funnel plot..……..…... ..…. ..…. ..…. .... ..…. ..…. ..…. ..…….….….18

**Figures 5-7.** Forest plot for complications sensitivity analysis..……...…………..……....….19-21

**Figure 7.** Hospital readmission funnel plot..…..……………………………………..……….….22

**Figure 8.** Forest plot for hospital readmission sensitivity analysis…..……...………..……....….23

**Figure 9.** Hospital length of stay (LOS) forest plot………….…………………..……….…..….24

**Figure 10.** Forest plot for hospital LOS sensitivity analysis..……..…………...………..……….25

**Table 1.** Preferred Reporting Items for Systematic Reviews and Meta-Analyses (PRISMA) 2020 Checklist

| **Section and Topic** | **Item#** | **Checklist item** | **Location where item is reported** |
| --- | --- | --- | --- |
| **TITLE** | | | |
| Title | 1 | Identify the report as a systematic review. | Title Page: 1 |
|  | | | |
| **ABSTRACT** | | | |
| Abstract | 2 | See the PRISMA 2020 for Abstracts checklist. | Summary: Page 4 |
|  | | | |
| **INTRODUCTION** | | | |
| Rationale | 4 | Describe the rationale for the review in the context of existing knowledge. | Manuscript: Page 6 |
| Objectives | 5 | Provide an explicit statement of the objective(s) or question(s) the review addresses. | Manuscript: Page 6 |
|  | | | |
| **METHODS** | | | |
| Eligibility criteria | 5 | Specify the inclusion and exclusion criteria for the review and how studies were grouped for the syntheses. | Manuscript: Page 6 |
| Information sources | 6 | Specify all databases, registers, websites, organisations, reference lists and other sources searched or consulted to identify studies. Specify the date when each source was last searched or consulted. | Manuscript: Page 6; Figure 1; Supplementary: Tables 2-3 |
| Search strategy | 7 | Present the full search strategies for all databases, registers and websites, including any filters and limits used. | Manuscript: Page 6-7;  Supplementary: Tables 2-3 |
| Selection process | 8 | Specify the methods used to decide whether a study met the inclusion criteria of the review, including how many reviewers screened each record and each report retrieved, whether they worked independently, and if applicable, details of automation tools used in the process. | Manuscript: Page 6-7 |
| Data collection process | 9 | Specify the methods used to collect data from reports, including how many reviewers collected data from each report, whether they worked independently, any processes for obtaining or confirming data from study investigators, and if applicable, details of automation tools used in the process. | Manuscript: Page 6-7 |
| Data items | 10a | List and define all outcomes for which data were sought. Specify whether all results that were compatible with each outcome domain in each study were sought (e.g. for all measures, time points, analyses), and if not, the methods used to decide which results to collect. | Manuscript: Page 6-7 |
|  | 10b | List and define all other variables for which data were sought (e.g. participant and intervention characteristics, funding sources). Describe any assumptions made about any missing or unclear information. | Manuscript: Pages 6-7 |
| Study risk of bias assessment | 11 | Specify the methods used to assess risk of bias in the included studies, including details of the tool(s) used, how many reviewers assessed each study and whether they worked independently, and if applicable, details of automation tools used in the process. | Manuscript: Pages 7 |
| Effect measures | 12 | Specify for each outcome the effect measure(s) (e.g. risk ratio, mean difference) used in the synthesis or presentation of results. | Manuscript: Page 7-8 |
| Synthesis methods | 13a | Describe the processes used to decide which studies were eligible for each synthesis (e.g. tabulating the study intervention characteristics and comparing against the planned groups for each synthesis (item #5)). | Manuscript: Pages 6-8 |
|  | 13b | Describe any methods required to prepare the data for presentation or synthesis, such as handling of missing summary statistics, or data conversions. | Manuscript: Pages 6-8 |
|  | 13c | Describe any methods used to tabulate or visually display results of individual studies and syntheses. | Manuscript: Pages 6-8 |
|  | 13d | Describe any methods used to synthesize results and provide a rationale for the choice(s). If meta-analysis was performed, describe the model(s), method(s) to identify the presence and extent of statistical heterogeneity, and software package(s) used. | Manuscript: Pages 6-8 |
|  | 13e | Describe any methods used to explore possible causes of heterogeneity among study results (e.g. subgroup analysis, meta-regression). | Manuscript: Pages 6-8 |
|  | 13f | Describe any sensitivity analyses conducted to assess robustness of the synthesized results. | Manuscript: Pages 6-7; Supplementary Figures |
| Reporting bias assessment | 14 | Describe any methods used to assess risk of bias due to missing results in a synthesis (arising from reporting biases). | Manuscript: Pages 7 |
| Certainty assessment | 15 | Describe any methods used to assess certainty (or confidence) in the body of evidence for an outcome. | N/A |
|  | | | |
| **RESULTS** | | | |
| Study selection | 16a | Describe the results of the search and selection process, from the number of records identified in the search to the number of studies included in the review, ideally using a flow diagram. | Manuscript: Page 8; Main Figure 1 |
|  | 16b | Cite studies that might appear to meet the inclusion criteria, but which were excluded, and explain why they were excluded. | Manuscript: Page 8, Main Figure 1, Supplementary Table 5 |
| Study characteristics | 17 | Cite each included study and present its characteristics. | Manuscript: Pages 8-9; Main Table 1 |
| Risk of bias in studies | 18 | Present assessments of risk of bias for each included study. | Manuscript: Pages 8-9;  Supplementary Table 4 |
| Results of individual studies | 19 | For all outcomes, present, for each study: (a) summary statistics for each group (where appropriate) and (b) an effect estimate and its precision (e.g. confidence/credible interval), ideally using structured tables or plots. | Manuscript: Pages 8-12, Main Figures 2-4;  Supplementary Figures |
| Results of syntheses | 20a | For each synthesis, briefly summarise the characteristics and risk of bias among contributing studies. | Manuscript: Pages 8-12 |
|  | 20b | Present results of all statistical syntheses conducted. If meta-analysis was done, present for each the summary estimate and its precision (e.g. confidence/credible interval) and measures of statistical heterogeneity. If comparing groups, describe the direction of the effect. | Manuscript: Pages 8-12; Main Figures 2-4;  Supplementary Figures |
|  | 20c | Present results of all investigations of possible causes of heterogeneity among study results. | Manuscript: Pages 8-12; Main Figures 2-4;  Supplementary Figures 2-6 |
|  | 20d | Present results of all sensitivity analyses conducted to assess the robustness of the synthesized results. | Manuscript: Pages 8-12; Figures 2-4;  Supplementary Figures |
| Reporting biases | 21 | Present assessments of risk of bias due to missing results (arising from reporting biases) for each synthesis assessed. | Manuscript: Pages 8-12; Supplementary Table 4 |
| Certainty of evidence | 22 | Present assessments of certainty (or confidence) in the body of evidence for each outcome assessed. | N/A |
|  | | | |
| **DISCUSSION** | | | |
| Discussion | 23a | Provide a general interpretation of the results in the context of other evidence. | Manuscript: Page 12-13 |
|  | 23b | Discuss any limitations of the evidence included in the review. | Manuscript: Page 13-15 |
|  | 23c | Discuss any limitations of the review processes used. | Manuscript: Page 14 |
|  | 23d | Discuss implications of the results for practice, policy, and future research. | Manuscript: Page 12-15 |
|  | | | |
| **OTHER INFORMATION** | | | |
| Registration and protocol | 24a | Provide registration information for the review, including register name and registration number, or state that the review was not registered. | Abstract;  Manuscript: Page 5 |
|  | 24b | Indicate where the review protocol can be accessed, or state that a protocol was not prepared. | Abstract;  Manuscript: Page 6 |
|  | 24c | Describe and explain any amendments to information provided at registration or in the protocol. | Manuscript: Page 7;  PROSPERO Registration Record |
| Support | 25 | Describe sources of financial or non-financial support for the review, and the role of the funders or sponsors in the review. | Title Page |
| Competing interests | 26 | Declare any competing interests of review authors. | Title Page |
| Availability of data, code and other materials | 27 | Report which of the following are publicly available and where they can be found: template data collection forms; data extracted from included studies; data used for all analyses; analytic code; any other materials used in the review. | Supplementary: Tables 2-3;  All data available upon reasonable request. |

**Table 2.** MEDLINE search strategy (inception to October 4th, 2023)

| **Line #** | **Search terms** | **Results** | **Comment** |
| --- | --- | --- | --- |
| 1 | physicians/ or allergists/ or anesthesiologists/ or cardiologists/ or dermatologists/ or endocrinologists/ or foreign medical graduates/ or gastroenterologists/ or general practitioners/ or geriatricians/ or hospitalists/ or nephrologists/ or neurologists/ or occupational health physicians/ or oncologists/ or radiation oncologists/ or ophthalmologists/ or osteopathic physicians/ or otolaryngologists/ or pathologists/ or pediatricians/ or neonatologists/ or physiatrists/ or physicians, family/ or physicians, primary care/ or pulmonologists/ or radiologists/ or rheumatologists/ or surgeons/ or neurosurgeons/ or "oral and maxillofacial surgeons"/ or orthopedic surgeons/ or urologists/ | 173701 | Physician terms |
| 2 | limit 1 to male | 41769 | Male limit |
| 3 | limit 1 to female | 45786 | Female limit |
| 4 | 2 and 3 | 37567 | Male and female physicians |
| 5 | ("male vs female" or "female vs male").ti,ab,kf. | 1482 | gender comparison textword trms |
| 6 | 1 and 5 | 30 | Physician gender comparison textword results |
| 7 | Physicians, Women/ or (male and female and (physician* or doctor* or surgeon*)).ti,ab,kf. | 19303 |  |
| 8 | 4 or 6 or 7 | 54477 | Physician gender terms |
| 9 | race factors/ or sex factors/ | 280379 |  |
| 10 | Sex Characteristics/ or ((gender or sex) adj2 characteristic*).ti,ab,kf. | 68003 |  |
| 11 | ((sex or gender) adj3 (concordance or discordance)).ti,ab,kf. | 436 |  |
| 12 | or/9-11 | 345114 | Gender or sex concordance/discordance terms |
| 13 | ((physician* or doctor* or surgeon* or resident*) adj2 (sex or gender or factor or factors or characteristic*)).ti,ab,kf. | 11396 | Physician gender textword terms |
| 14 | "length of stay"/ or patient admission/ or patient discharge/ or patient readmission/ or death/ or cause of death/ or mortality/ or hospital mortality/ or survival rate/ or fatal outcome/ or (mortality or readmission or death* or fatal or fatalit*).ti,ab,kf. or mo.fs. | 2482506 | Mortality terms |
| 15 | 8 and 12 and 14 | 314 | Base set 1 |
| 16 | 13 and 14 | 1433 | Base set 2 |
| 17 | 15 or 16 | 1720 | Final results |
| 18 | limit 17 to english language | 1650 | Language limit |

**Table 3.** EMBASE search strategy (inception to October 4th, 2023)

| **Line #** | **Search terms** | **Results** | **Comment** |
| --- | --- | --- | --- |
| 1 | physician/ or andrologist/ or anesthesiologist/ or exp cardiologist/ or exp dermatologist/ or diabetologist/ or emergency physician/ or endocrinologist/ or epileptologist/ or foreign physician/ or gastroenterologist/ or general practitioner/ or geriatrician/ or gerontologist/ or exp gynecologist/ or exp hematologist/ or hepatologist/ or hospital physician/ or immunologist/ or infectious disease specialist/ or intensivist/ or internist/ or medical geneticist/ or neonatologist/ or nephrologist/ or neurologist/ or exp obstetrician/ or occupational physician/ or exp oncologist/ or ophthalmologist/ or exp orthopedic specialist/ or osteopathic physician/ or otolaryngologist/ or exp pathologist/ or exp pediatrician/ or phlebologist/ or physiatrist/ or podiatrist/ or psychiatrist/ or pulmonologist/ or exp radiologist/ or rheumatologist/ or exp surgeon/ or exp urologist/ or vaccinologist/ or exp venereologist/ | 970599 | Physician terms |
| 2 | limit 1 to male | 340446 | Male limit |
| 3 | limit 1 to female | 374542 | Female limit |
| 4 | 2 and 3 | 276776 | Male and female physicians |
| 5 | ("male vs female" or "female vs male").ti,ab,kf. | 2807 | gender comparison textword trms |
| 6 | 1 and 5 | 187 | Physician gender comparison textword results |
| 7 | female physician/ or (male and female and (physician* or doctor* or surgeon*)).ti,ab,kf. | 29975 |  |
| 8 | 4 or 6 or 7 | 294367 | Physician gender terms |
| 9 | femininity/ or masculinity/ or race/ | 92900 |  |
| 10 | sex difference/ or "gender and sex"/ or sex factor/ or sexual characteristics/ | 447683 |  |
| 11 | ((gender or sex) adj2 characteristic*).ti,ab,kf. | 11597 |  |
| 12 | ((sex or gender) adj3 (concordance or discordance)).ti,ab,kf. | 593 |  |
| 13 | or/9-12 | 556501 | Gender or sex concordance/discordance terms |
| 14 | ((physician* or doctor* or surgeon* or resident*) adj2 (sex or gender or factor or factors or characteristic*)).ti,ab,kf. | 15239 | Physician gender textword terms |
| 15 | hospital admission/ or hospital discharge/ or hospital readmission/ or hospitalization/ | 946533 |  |
| 16 | death/ or "cause of death"/ or fatality/ or heart death/ or lethality/ | 556501 |  |
| 17 | mortality/ or hospital mortality/ or in-hospital mortality/ or mortality rate/ or case fatality rate/ or crude mortality rate/ or infection fatality rate/ or surgical mortality/ or survival rate/ | 1350085 |  |
| 18 | (mortality or readmission or death* or fatal or fatalit*).ti,ab,kf. | 2809976 |  |
| 19 | or/15-18 | 3959163 | Mortality terms |
| 20 | 4 and 13 and 19 | 2148 | Base set 1 |
| 21 | 14 and 19 | 2406 | Base set 2 |
| 22 | 20 or 21 | 4468 | Final results |
| 23 | limit 22 to (chapter or conference abstract or conference paper or "conference review") | 1764 | Conference abstracts |
| 24 | 22 not 23 [***conference abstracts removed****] | 2704 | Conference abstracts removed |
| 25 | limit 24 to english language | 2608 | Language limit |

**Table 4.** ROBINS-I risk of bias ratings

| **Study Identifier** | **Confounding** | **Selection of participants** | **Classification of interventions** | **Deviations from intended interventions** | **Missing data** | **Measurement of outcomes** | **Selective reporting** | **Overall risk of bias rating** |
| --- | --- | --- | --- | --- | --- | --- | --- | --- |
| Becker, Siry-Bove, Shelton, et al. | Critical | Moderate | Moderate | Moderate | Moderate | Moderate | Moderate | Critical |
| Bouchgoul, Deneux-Tharaux, Georget, et al. | Moderate | Moderate | Low | Low | Low | Low | Moderate | Moderate |
| Blohm, Sandblom, Enochsson, et al. | Severe | Moderate | Moderate | Moderate | Low | Moderate | Moderate | Severe |
| Berg, Hurtig & Steinsbekk | Severe | Moderate | Moderate | Moderate | Severe | Moderate | Moderate | Severe |
| Chai, Chen, Lin & Lin | Severe | Moderate | Moderate | Moderate | Moderate | Moderate | Moderate | Severe |
| Chapman, Zmistowski, Votta, et al. | Severe | Low | Moderate | Moderate | Moderate | Severe | Severe | Severe |
| Dwyer & Kalın | Critical | Low | Moderate | Moderate | Moderate | Moderate | Moderate | Critical |
| Dziewierz, Vogel, Zdzierak, et al. | Severe | Low | Moderate | Moderate | Moderate | Moderate | Moderate | Severe |
| Etherington, Boet, Chen, et al. | Moderate | Low | Moderate | Moderate | Moderate | Moderate | Moderate | Moderate |
| Flodin J, Juthberg R, Edman G & Ackermann | Critical | Moderate | Moderate | Moderate | Low | Moderate | Moderate | Critical |
| Greenwood, Carnaha, Huang | Moderate | Low | Moderate | Moderate | Moderate | Moderate | Moderate | Moderate |
| Haubitz-Eshchelbach, Mirsada, Sebastian, et al. | Severe | Low | Low | Low | Low | Low | Moderate | Severe |
| Ho, Kuo, Tsai, et al. | Moderate | Low | Moderate | Moderate | Moderate | Moderate | Moderate | Moderate |
| Jerath, Satkunasivam, Kaneshwaran, et al. | Moderate | Low | Moderate | Moderate | Moderate | Moderate | Moderate | Moderate |
| Jolback, Rogmark, Bedeschi, et al. | Moderate | Low | Moderate | Moderate | Moderate | Moderate | Moderate | Moderate |
| Kobylianskii, Murji, Matelski, et al. | Moderate | Low | Moderate | Moderate | Low | Moderate | Moderate | Moderate |
| Mazilescu LI, Bernheim I, Treckmann J, et al. | Critical | Moderate | Moderate | Moderate | Low | Moderate | Moderate | Critical |
| Meier, Yang, Liu, et al. | Critical | Low | Moderate | Moderate | Moderate | Moderate | Moderate | Critical |
| Nakayama, Morita, Fujiwara & Komuro | Severe | Low | Low | Moderate | Moderate | Moderate | Moderate | Severe |
| O’Neill, Lanska & Hartz | Severe | Low | Moderate | Moderate | Moderate | Moderate | Moderate | Severe |
| Okoshi, Endo & Nomura | Severe | Low | Low | Moderate | Low | Moderate | Moderate | Severe |
| Rifkin, Holmboe, Scherer, et al. | Moderate | Moderate | Moderate | Moderate | Moderate | Moderate | Moderate | Moderate |
| Sagy, Fuchs, Mizrakli, et al. | Severe | Severe | Moderate | Moderate | Moderate | Moderate | Moderate | Severe |
| Sergeant, Saha, Shin et al. | Moderate | Low | Moderate | Moderate | Moderate | Moderate | Moderate | Moderate |
| Sharoky, Sellers, Keele, et al. | Severe | Low | Moderate | Moderate | Severe | Moderate | Moderate | Severe |
| Shen, Li, Wu & Yang | Moderate | Severe | Moderate | Moderate | Moderate | Moderate | Moderate | Severe |
| Sun, Boet, Chan, et al. | Severe | Low | Moderate | Moderate | Severe | Moderate | Moderate | Severe |
| Tsugawa, Jena, Figueroa, et al. | Moderate | Severe | Moderate | Moderate | Severe | Moderate | Moderate | Severe |
| Tsugawa, Jena, Orav, et al. | Moderate | Low | Moderate | Moderate | Low | Moderate | Moderate | Moderate |
| Wallis, Jerath, Coburn, et al. | Moderate | Low | Moderate | Moderate | Moderate | Moderate | Moderate | Moderate |
| Wallis, Jerath, Kaneshwaran, et al. | Moderate | Low | Moderate | Moderate | Moderate | Moderate | Moderate | Moderate |
| Wallis, Jerath, Satkunasivam, et al. | Moderate | Low | Moderate | Moderate | Moderate | Moderate | Moderate | Moderate |
| Wallis, Ravi, Coburn, et al. | Moderate | Low | Moderate | Moderate | Moderate | Moderate | Moderate | Moderate |
| Wu, Wu & Weng | Moderate | Low | Moderate | Moderate | Moderate | Moderate | Moderate | Severe |
| Yelavarthy, Seth, Pielsticker, et al. | Moderate | Low | Moderate | Moderate | Moderate | Moderate | Moderate | Moderate |

**Table 5.** List of excluded studies during full-text screening

| **Citation** | **Exclusion Reason** |
| --- | --- |
| Etherington N, Deng M, Boet S, et al. Impact of physician’s sex/gender on processes of care, and clinical outcomes in cardiac operative care: a systematic review. BMJ Open 2020; 10: e037139. | Wrong study design - Review |
| Woodward M. Cardiovascular Disease and the Female Disadvantage. Int J Environ Res Public Health 2019; 16. DOI:10.3390/ijerph16071165. | Wrong study design - Review |
| Lau ES, Hayes SN, Volgman AS, et al. Does Patient-Physician Gender Concordance Influence Patient Perceptions or Outcomes? J Am Coll Cardiol 2021; 77: 1135–8. | Wrong study design - Review |
| Hunziker S. Female or Male Team Leader During Cardio Pulmonary Resuscitation: Does It Really Matter? Crit. Care Med. 2019; 47: 144–6. | Wrong study design - Editorial |
| Collins J, Abbass IM, Harvey R, et al. Predictors of all-cause 30 day readmission among Medicare patients with type 2 diabetes. Curr Med Res Opin 2017; 33: 1517–23. | Wrong exposure - Did not investigate physician sex/gender |
| Alasmar M, McKechnie I, Chaparala RPC. Emergency surgery for hiatus hernias: does technique affect outcomes? A single-centre experience. Updates Surg 2023; 75: 1227–33. | Wrong exposure - Did not investigate physician sex/gender |
| Lai JC-Y, Chen H-H, Huang S-M, et al. In-hospital complications of vaginal versus laparoscopic-assisted benign hysterectomy among older women: a propensity score-matched cohort study. Menopause 2016; 23: 1233–8. | Wrong exposure - Did not investigate physician sex/gender |
| Sapci I, Velazco JS, Xhaja X, et al. Factors associated with noncomplete mesorectal excision following surgery for rectal adenocarcinoma. Am J Surg 2019; 217: 465–8. | Wrong exposure - Did not investigate physician sex/gender |
| Svedahl ER, Pape K, Austad B, et al. Effects of GP characteristics on unplanned hospital admissions and patient safety. A 9-year follow-up of all Norwegian out-of-hours contacts. Fam Pract 2022; 39: 381–8. | Wrong exposure - Did not investigate physician sex/gender |
| Chun DS, Cook RW, Weiner JA, et al. Can Surgeon Demographic Factors Predict Postoperative Complication Rates After Elective Spinal Fusion? Clin Spine Surg 2018; 31: 93–7. | Wrong exposure - Underpowered to investigate physician sex/gender |
| Patient gender affects skin cancer screening practices and attitudes among veterans. 2014; published online Jan 10. https://sma.org/southern-medical-journal/article/patient-gender-affects-skin-cancer-screening-practices-and-attitudes-among-veterans/ (accessed Nov 7, 2023). | Wrong exposure - Did not investigate physician sex/gender |
| Garcia-Ochoa C, Feldman LS, Nguan C, et al. Perioperative Complications During Living Donor Nephrectomy: Results From a Multicenter Cohort Study. Can J Kidney Health Dis 2019; 6: 2054358119857718. | Wrong exposure - Did not investigate physician sex/gender |
| Jackson RE, Anderson W, Peacock WF IV, Vaught L, Carley RS, Wilson AG: Effect of a patient's sex on timing of thrombolytic therapy. *Ann Emerg Med* 1996; 27: 8-15 | Wrong exposure - Did not investigate physician sex/gender |
| Jerant A, Bertakis KD, Fenton JJ, Franks P. Gender of physician as the usual source of care and patient health care utilization and mortality. J Am Board Fam Med 2013; 26: 138–48. | Wrong exposure - Did not consider much care respondents received, if at all, from their primary care physician in the study |
| Huang K-C, Lin Y-R, Syue Y-J, Kung C-T, Chiu I-M, Li C-J. Comparison of Clinical Practice in the Emergency Department: Female Versus Male Emergency Physicians. Am J Med Sci 2018; 355: 215–9. | Wrong outcome - No outcomes of interest |
| Dahrouge S, Seale E, Hogg W, et al. A Comprehensive Assessment of Family Physician Gender and Quality of Care: A Cross-Sectional Analysis in Ontario, Canada. Med Care 2016; 54: 277–86. | Wrong outcome - No outcomes of interest |
| AlAshqar A, Wildey B, Yazdy G, Goktepe ME, Kilic GS, Borahay MA. Predictors of same-day discharge after minimally invasive hysterectomy for benign indications. Int J Gynaecol Obstet 2022; 158: 308–17. | Wrong outcome - No outcomes of interest |
| Lai H-Y, Hwang S-J, Chen Y-C, Chen T-J, Lin M-H, Chen L-K. Prevalence of the prescribing of potentially inappropriate medications at ambulatory care visits by elderly patients covered by the Taiwanese National Health Insurance program. Clin Ther 2009; 31: 1859–70. | Wrong outcome - No outcomes of interest |
| Yee LM, Miller ES. Association of Obstetrician Gender With Obstetric Interventions and Outcomes. Obstet Gynecol 2018; 132: 79–84. | Wrong outcome - No outcomes of interest |

**Figure 1.** Mortality funnel plot

**
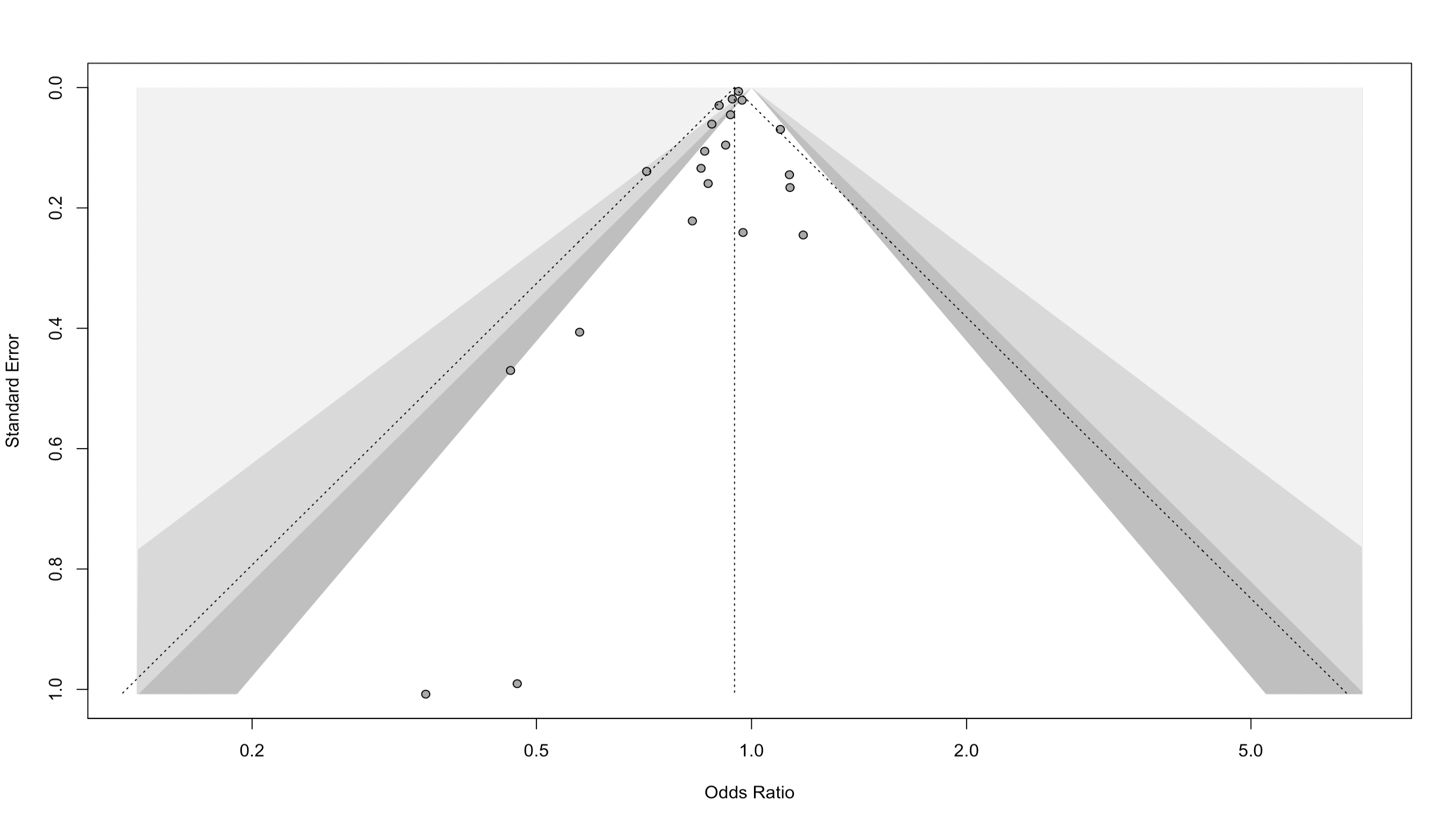
**

Darkest, dark, and light grey contours correspond to P < 0.1, 0.05, 0.01, respectively. Egger’s test did not reveal significant publication bias (P_Egger_=0.08).

**Figure 2.** Forest plot for mortality sensitivity analysis excluding Jerath et al.

*
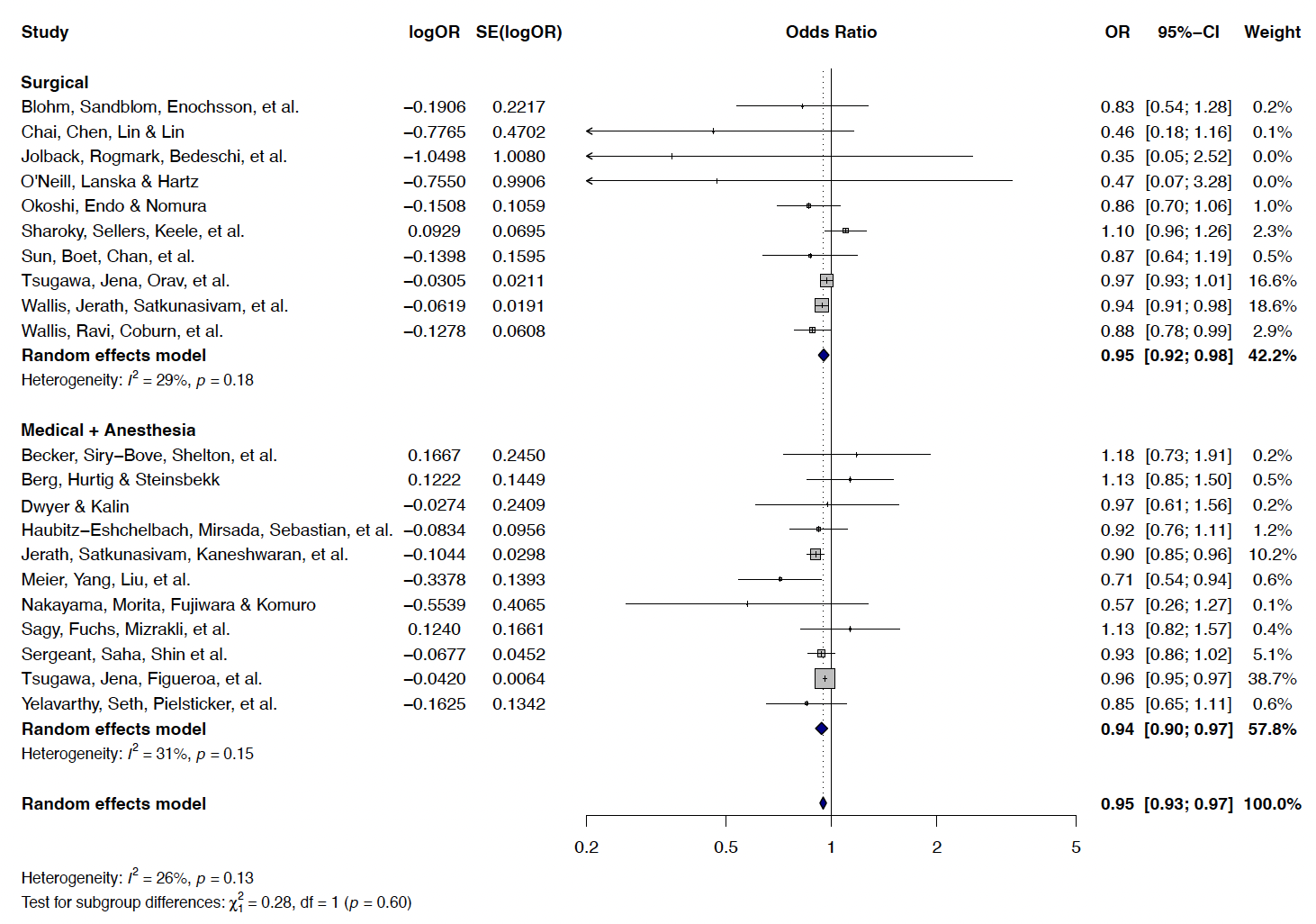
*

*Abbreviations: OR – Odds ratio; SE – Standard error; CI – Confidence interval*

**Figure 3.** Forest plot for mortality subgroup analysis stratified by North America versus Other Continent location

**
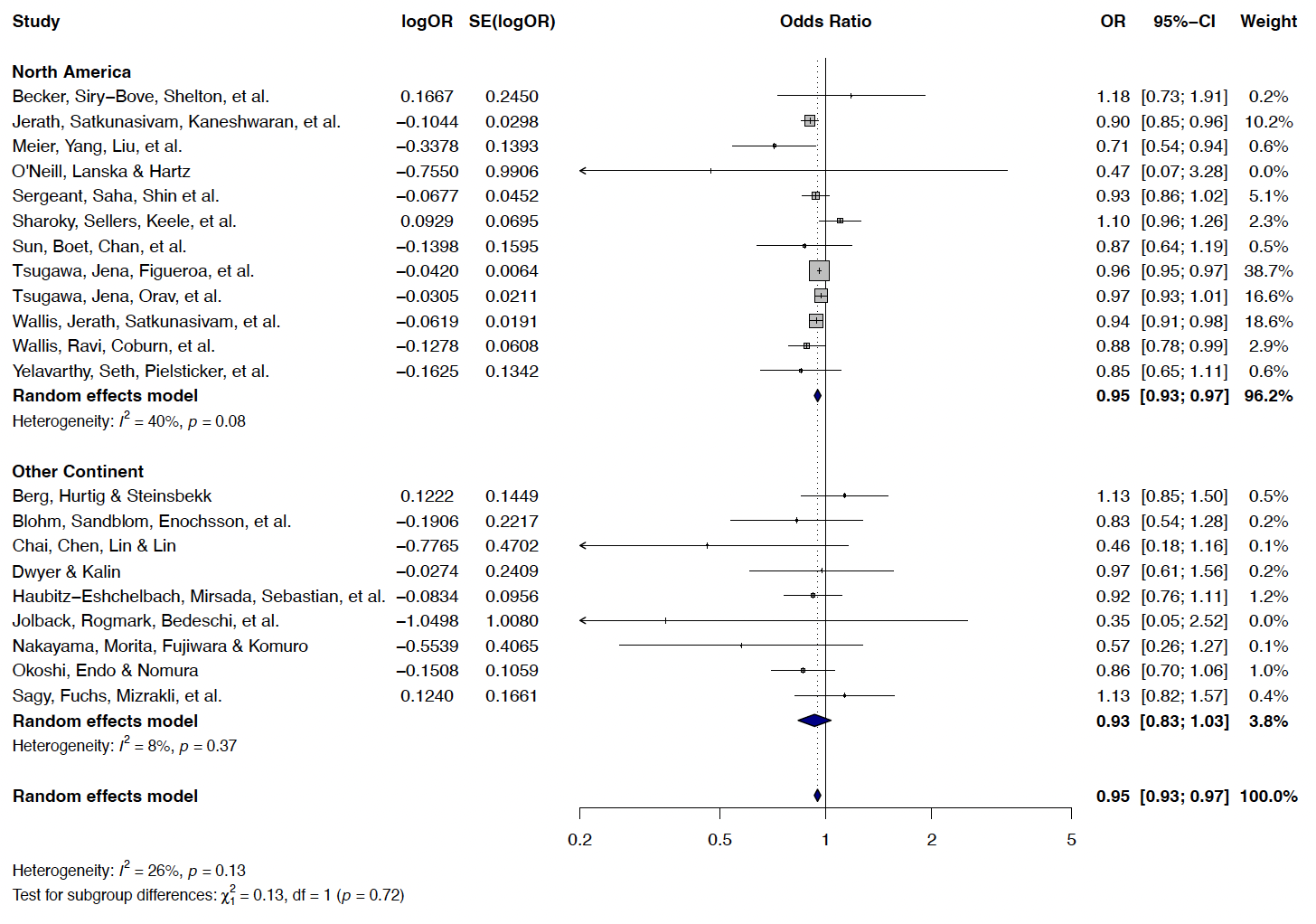
**

*Abbreviations: OR – Odds ratio; SE – Standard error; CI – Confidence interval*

**Figure 4.** Complications funnel plot

**
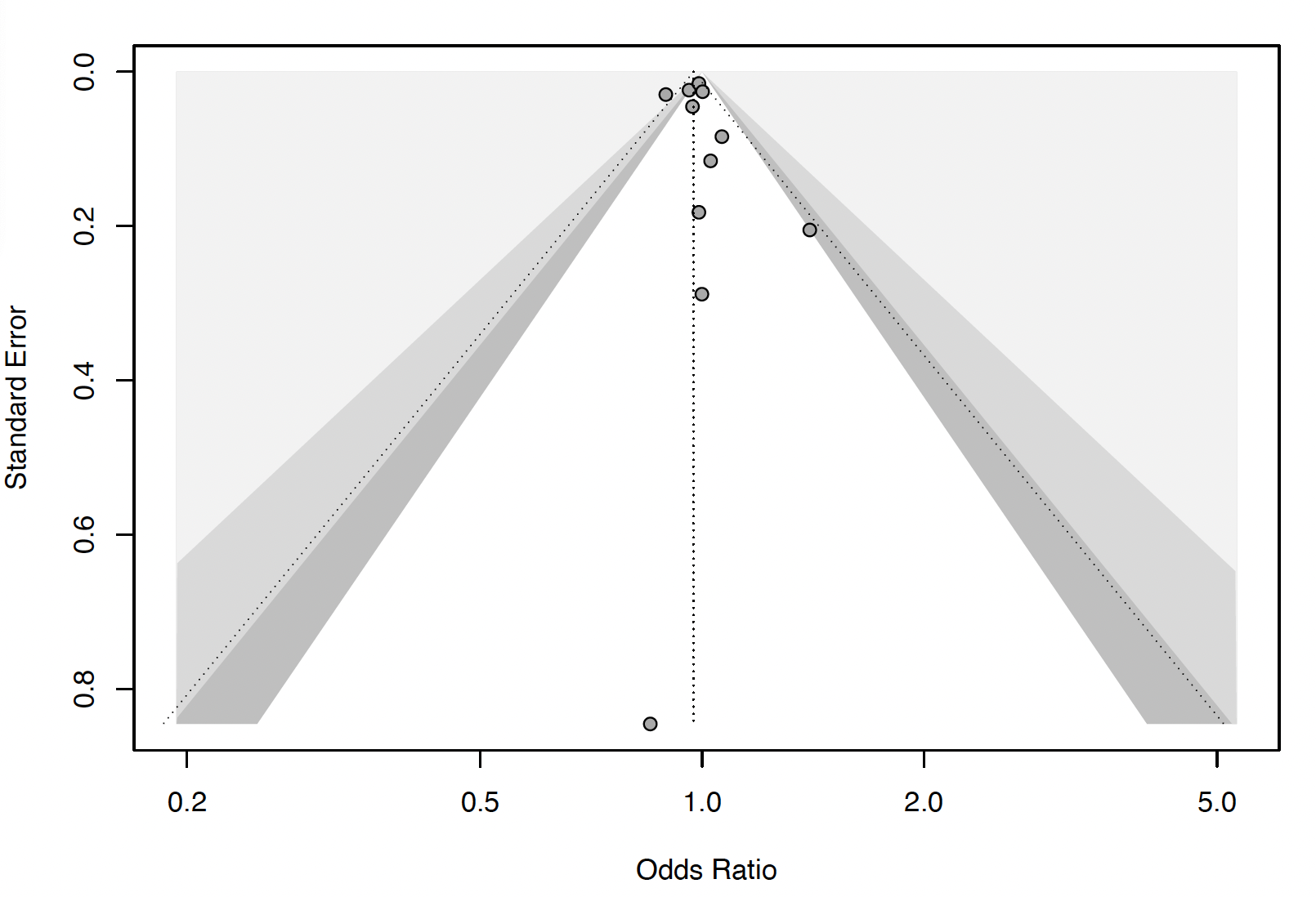
**

Darkest, dark, and light grey contours correspond to P < 0.1, 0.05, 0.01, respectively. Egger’s test did not reveal significant publication bias (P_Egger_=0.62).

**Figure 5.** Forest plot for complications sensitivity analysis excluding Jerath et al.


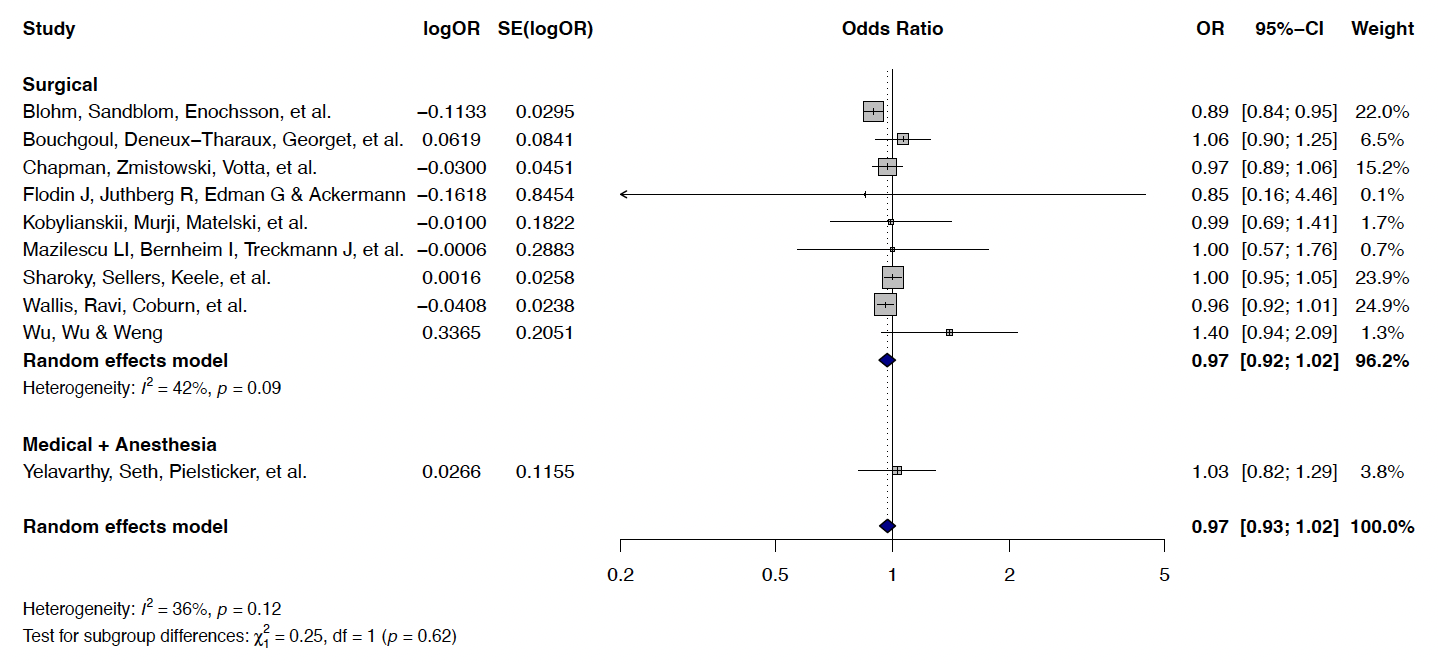


*Abbreviations: OR – Odds ratio; SE – Standard error; CI – Confidence interval*

**Figure 6.** Forest plot for complications sensitivity analysis excluding Chapman et al.


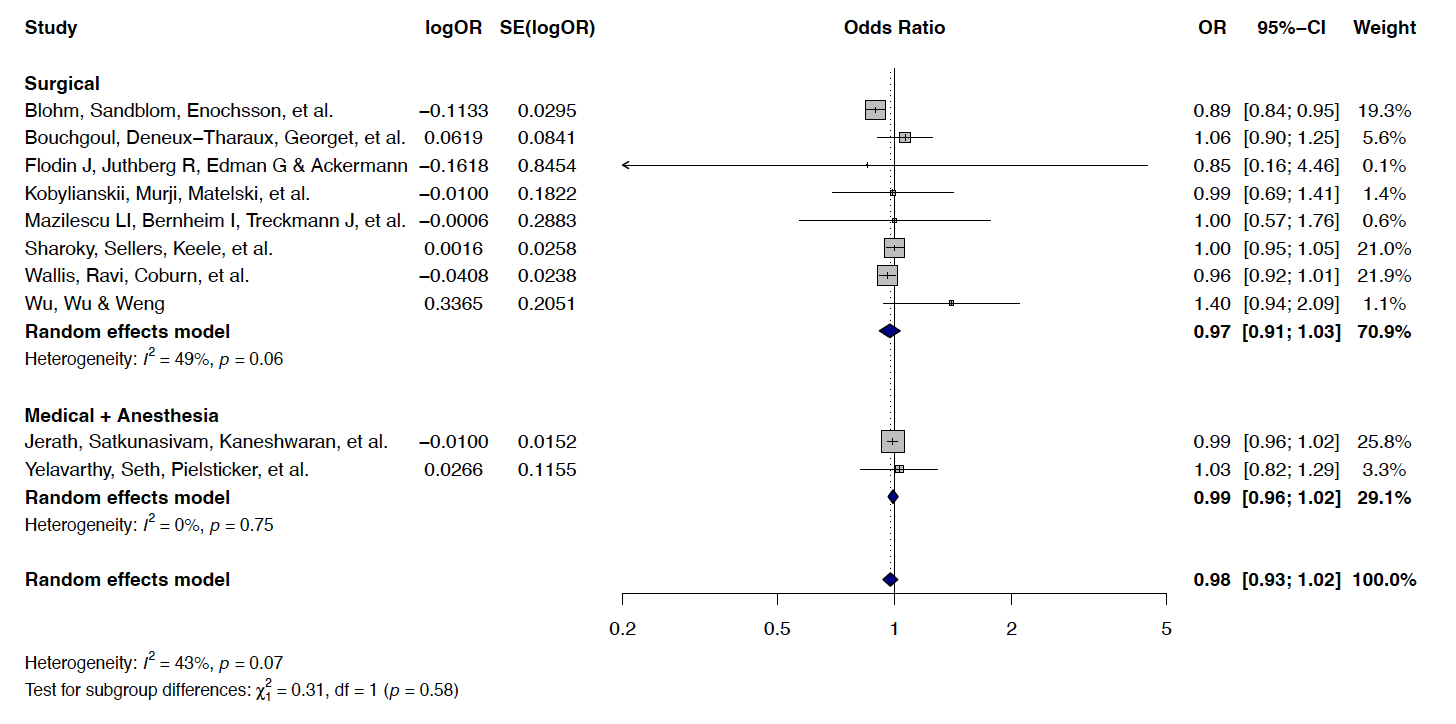


*Abbreviations: OR – Odds ratio; SE – Standard error; CI – Confidence interval*

**Figure 7.** Forest plot for complications sensitivity analysis using unadjusted proportions


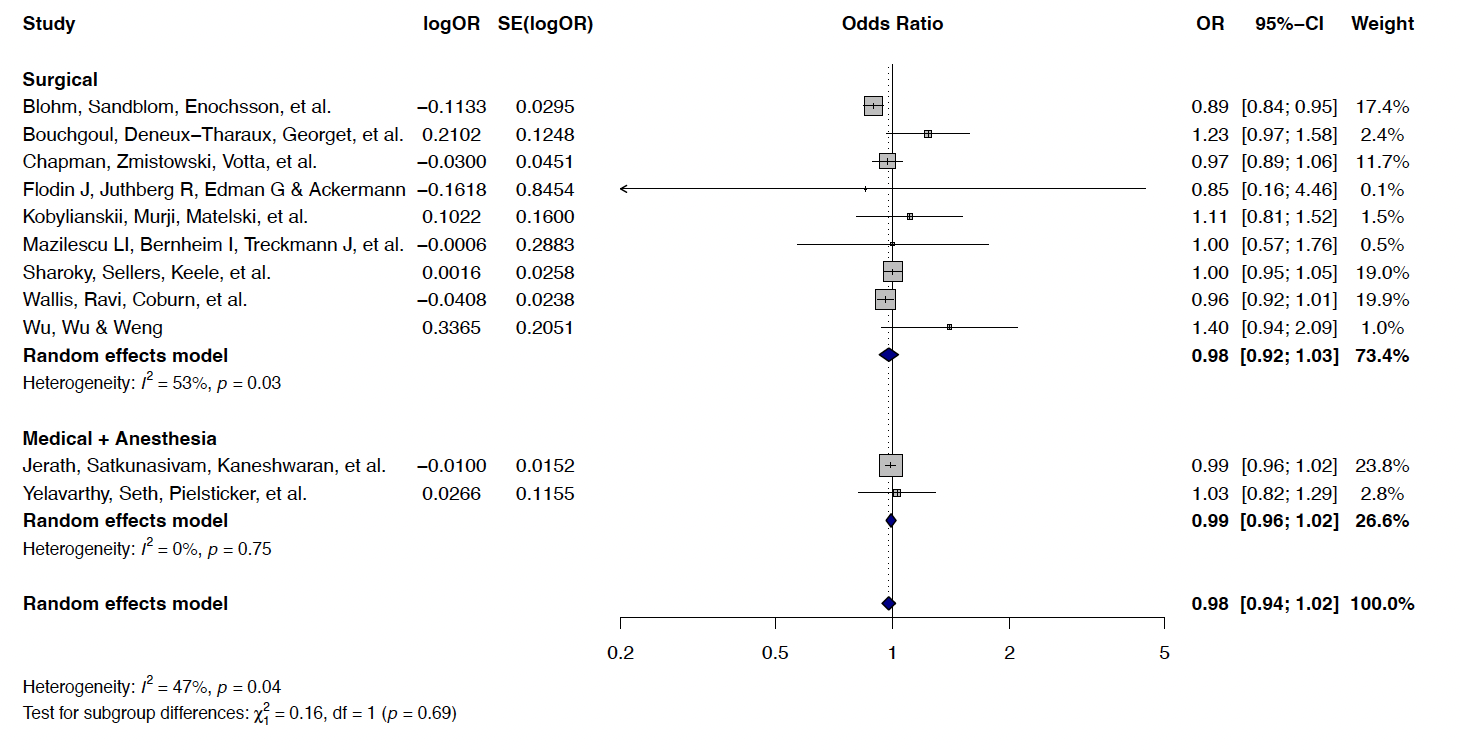


*Abbreviations: OR – Odds ratio; SE – Standard error; CI – Confidence interval*

**Figure 8.** Hospital readmission funnel plot

**
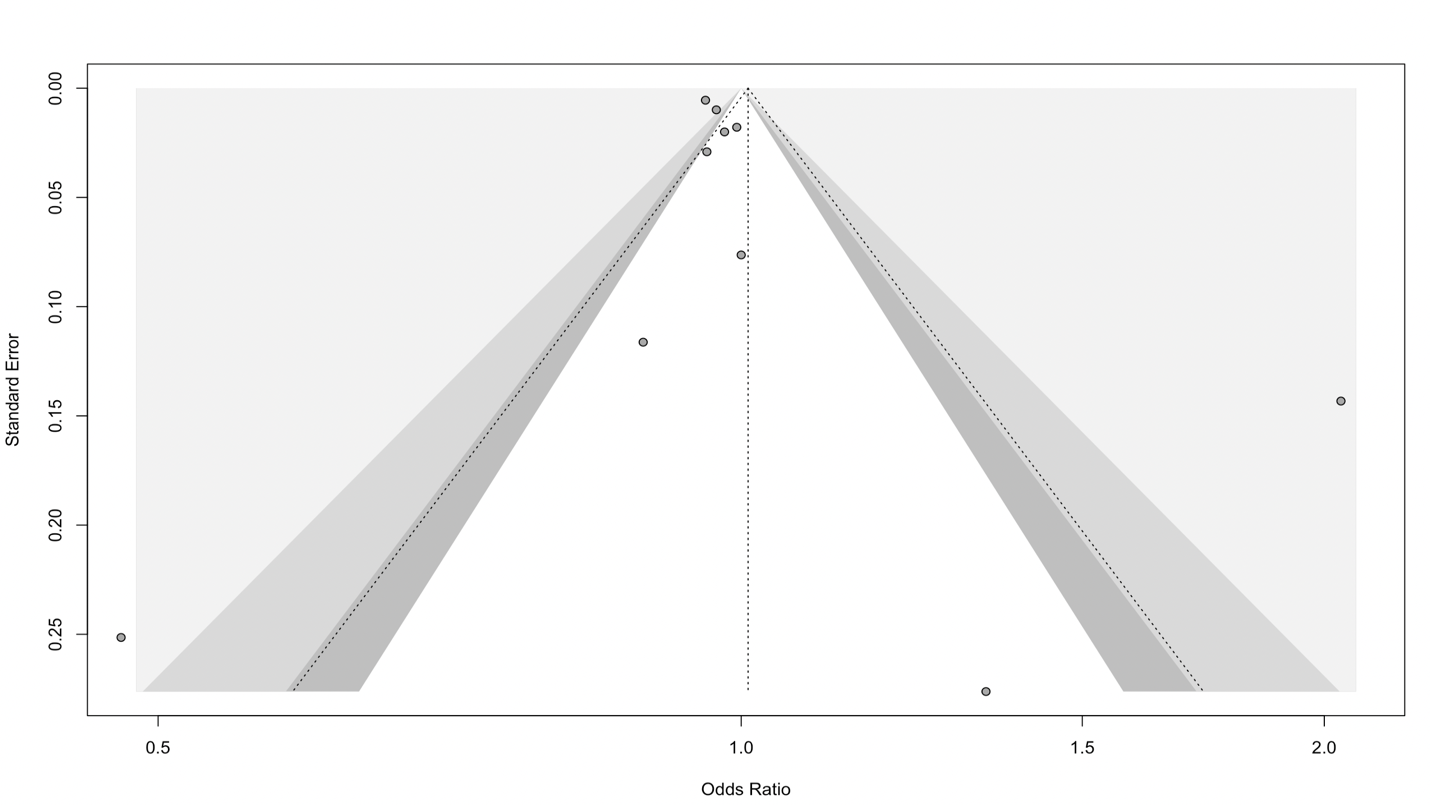
**

Darkest, dark, and light grey contours correspond to P < 0.1, 0.05, 0.01, respectively. Egger’s test did not reveal significant publication bias (P_Egger_=0.36).

**Figure 9.** Forest plot for hospital readmission sensitivity analysis excluding Jerath et al.


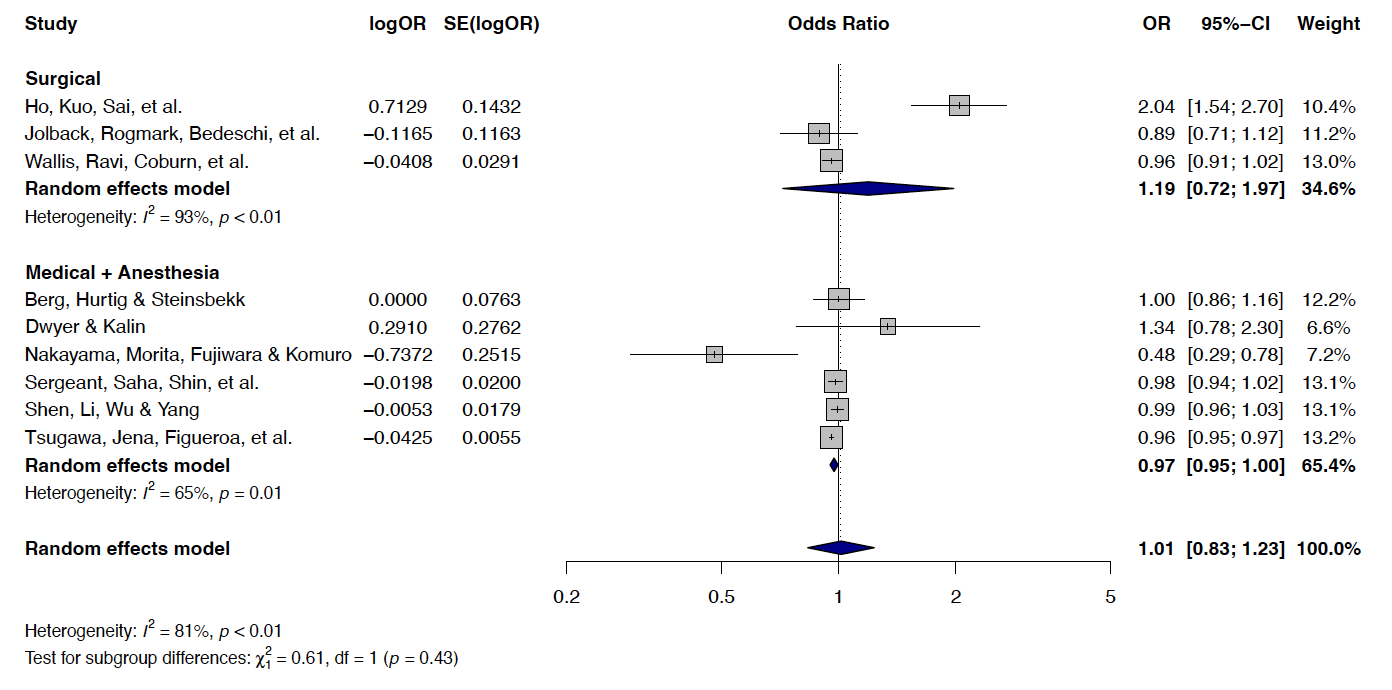


*Abbreviations: OR – Odds ratio; SE – Standard error; CI – Confidence interval*

**Figure 10.** Hospital length of stay (LOS) forest plot


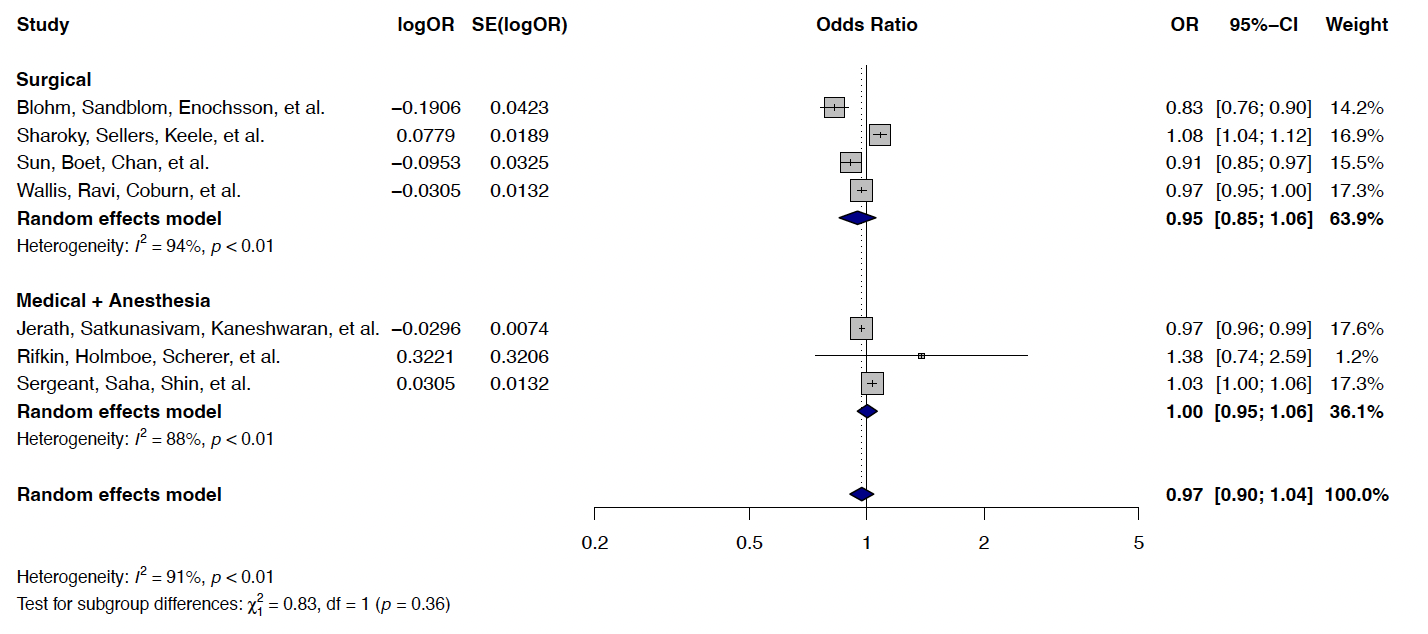


*Abbreviations: OR – Odds ratio; SE – Standard error; CI – Confidence interval*

**Figure 11.** Forest plot for hospital length of stay (LOS) sensitivity analysis excluding Jerath et al.

*
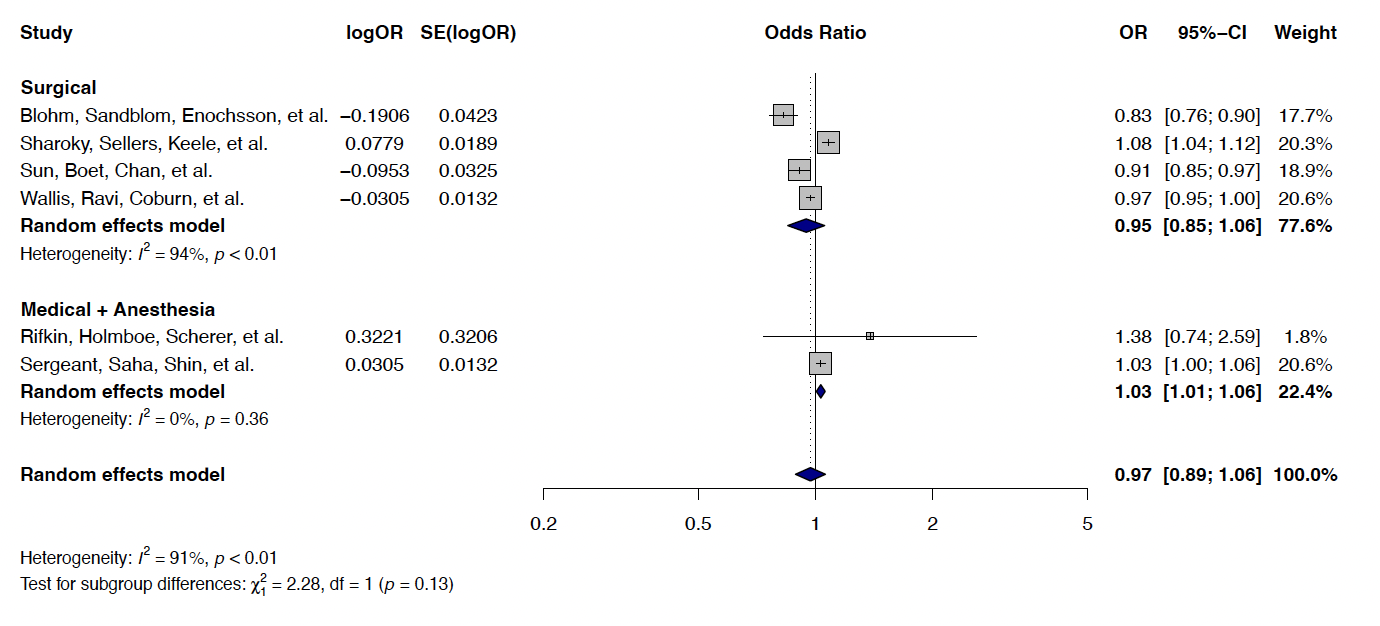
Abbreviations: OR – Odds ratio; SE – Standard error; CI – Confidence interval*
