# Supplementary material for: The association between physician sex and patient outcomes: a systematic review and meta-analysis
Source: BMC Health Serv Res. 2025 Jan 17;25:93. doi: 10.1186/s12913-025-12247-1 (PMC11740500; doi:10.1186/s12913-025-12247-1)
Supplement: Supplementary file 2 — Supplementary Material 2. [file 12913_2025_12247_MOESM2_ESM.docx]

**Electronic Supplementary Materials 2**

**The association between physician sex and patient outcomes: A systematic review and meta-analysis**

Kiyan Heybati BHSc^1^, Ashton Chang MD^2^, Hodan Mohamud BSc^3^, Raj Satkunasivam MD MSc^4,5,6^, Natalie Coburn MD MPH^7^, Arghavan Salles MD PhD^8^, Yusuke Tsugawa MD PhD^9,10^, Ryo Ikesu MD,^11^ Natsumi Saka MD PhD^12,13^, Allan S. Detsky MD PhD^14,15,16^, Dennis T Ko MD MSc^15,16,17,18^, Heather Ross MD MHSc^19^, Mamas A. Mamas MD DPhil^20^, Angela Jerath MD MSc^2, 15, 17, 18^*****, Christopher JD Wallis MD PhD^21,22^*

**Equal contribution senior authors*

**ADDITIONAL RESULTS**

*All-cause mortality*

Across individual studies, in separate patient cohorts with adjustment for baseline covariates, Wallis et al.[^39^](https://paperpile.com/c/3sOdUZ/UR4Ub) (n= 2,732,565) and Wallis et al.[^28^](https://paperpile.com/c/3sOdUZ/8AQHi) (n= 104,630) enrolled patients undergoing surgery and found significantly lower mortality when comparing patients of female versus male physicians (adjusted OR [aOR] 0·94 (95% CI: 0·91 to 0·98), and aOR 0·88 (95% CI: 0·78 to 0·99), respectively). Similarly, across the medical/anesthesia cohort, Jerath et al.[^25^](https://paperpile.com/c/3sOdUZ/RUJ9u) (n= 1,165,711), and Tsugawa et al.[^6^](https://paperpile.com/c/3sOdUZ/sU2SN) (n=1,283,621) reported significantly lower mortality among patients treated by female physicians (aOR 0·90; 95% CI: 0·85 to 0·96, and aOR 0·96; 95% CI: 0·95 to 0·97, respectively). Notably, these four studies represent the largest cohorts included in this review. At the same time, Meier et al.[^47^](https://paperpile.com/c/3sOdUZ/DRCNu) (n =1,082) also found significantly lower unadjusted mortality and, following adjustment, they reported higher odds of survival among patients of female physicians (aOR 1·53; 95% CI: 1·15 to 2·02). However, the remaining 15 studies did not identify significant differences in mortality based on physician sex (**Figure 2**).

*Complications*

Across individual studies, Blohm et al.[^40^](https://paperpile.com/c/3sOdUZ/UlLBx) (n=146,416) found significantly lower odds of complications among patients of female versus male surgeons (aOR 0·89; 95% CI: 0·84 to 0·94; **Figure 3**), while the remaining studies did not find a significant association.

*Hospital readmission*

Across individual studies, three[^6,25,33^](https://paperpile.com/c/3sOdUZ/DfjhL+sU2SN+RUJ9u) found a significant decrease in readmission for patients of female physicians while one[^37^](https://paperpile.com/c/3sOdUZ/TUCOW) reported significantly higher rates (**Figure 4**). Jerath et al.[^25^](https://paperpile.com/c/3sOdUZ/RUJ9u) (n=1,165,711), Nakayama et al.[^33^](https://paperpile.com/c/3sOdUZ/DfjhL) (n=9,544) and Tsugawa et al.[^6^](https://paperpile.com/c/3sOdUZ/sU2SN) (n=1,249,210) found significantly lower odds of hospital readmission when comparing patients of female versus male physicians (aOR 0·97; 95% CI: 0·95 to 0·99, aOR 0·48; 95% CI: 0·28 to 0·78, and aOR 0·96; 95% CI: 0·95 to 0·97, respectively). Conversely, Ho et al.[^37^](https://paperpile.com/c/3sOdUZ/TUCOW) (n=7,427) examined 180-day readmission rates and reported significantly higher odds of readmission across patients treated by female versus male ophthalmologists (aOR 2·04; 95% CI: 1·54 to 2·70).

*Hospital length of stay (LOS)*

Across individual studies, Sun et al.[^29^](https://paperpile.com/c/3sOdUZ/O2CUw) (n=79,862), Wallis et al.[^28^](https://paperpile.com/c/3sOdUZ/8AQHi) (n=104,630), and Blohm et al.[^40^](https://paperpile.com/c/3sOdUZ/UlLBx) (n=147,304) found significantly shorter hospital LOS when comparing patients of female versus male surgeons (aOR 0·91 (95% CI: 0·85 to 0·97), aOR 0·97 (95% CI: 0·95 to 1·00), and aOR 0·83 (95% CI: 0·76 to 0·90), respectively). Similarly, Jerath et al.[^25^](https://paperpile.com/c/3sOdUZ/RUJ9u) (n=1,165,711) found significantly lower LOS among patients of female anesthesiologists (aOR 0·97; 95% CI: 0·96 to 0·99). Berg et al.[^43^](https://paperpile.com/c/3sOdUZ/6IFBD) (n=11,059) reported a significantly lower LOS among patients of on-call female physicians at the time of admission (adjusted coefficient -0·23 days (95% CI: -0·42 to -0·04 days)). Conversely, Sharoky et al.[^10^](https://paperpile.com/c/3sOdUZ/StXE1) (n=45,838) and Sergeant et al.[^7^](https://paperpile.com/c/3sOdUZ/lx4rS) (n=171,625) reported significantly higher hospital LOS among patients managed by female versus male physicians (aOR 1·08 (95% CI: 1·04 to 1·12), and aOR 1·03 (95% CI: 1·00 to 1·06), respectively).

*Physician sex concordance*

Wallis et al.[^2^](https://paperpile.com/c/3sOdUZ/mW3T3) (n=1,320,108) demonstrated that sex discordance was associated with higher odds of complications (aOR 1·09; 95% CI: 1·07 to 1·11). In subgroup analyses, female patients had higher odds of complications with a sex-discordant surgeon compared to a sex-concordant surgeon (aOR 1·16; 95% CI: 1·11 to 1·22); however, males had similar odds of complications regardless of surgeon sex (aOR 1·02; 95% CI: 0·98 to 1·06). Yelvarathy et al.[^8^](https://paperpile.com/c/3sOdUZ/eSWY3) (n=239,422) assessed major bleeding, need for transfusions, and acute kidney injury as complications following percutaneous coronary intervention and found no significant differences, regardless of proceduralist-patient sex concordance. Similarly, Dziewierz et al.[^44^](https://paperpile.com/c/3sOdUZ/pbTbe) (n=581,744) also found no significant associations.

Wallis et al.[^2^](https://paperpile.com/c/3sOdUZ/mW3T3) (n=1,320,108) also reported on readmission and hospital LOS. Sex discordance was associated with higher odds of hospital readmission (aOR 1·02; 95% CI: 0·98 to 1·07) and hospital LOS (aOR 1·11; 95% CI: 1·06 to 1·15). Female patients with a male physician experienced a significantly higher number of readmissions (aOR 1·11; 95% CI: 1·04 to 1·19) and longer hospital LOS (aOR 1·20; 95% CI: 1·11 to 1·30) compared to those cared for by a female physician. However, male patients with a female physician experienced a comparable number of readmissions (aOR 0·94; 95% CI: 0·88 to 1·00) and no significant difference in hospital LOS (aOR 1·02; 95% CI: 0·96 to 1·08) compared to those cared for by a male physician. Greenwood et al.[^11^](https://paperpile.com/c/3sOdUZ/kdGez) (n=581,845) found that female patients treated by male physicians experienced longer hospital LOS compared to male patients with male physicians (matched n=119,304; mean [SD] 7·386 [9·333] versus 6·841 [7·992] days). This discrepancy in hospital LOS was less pronounced among male and female patients with female physicians (matched n=15,122; male patient/female physician 6·907 [8·607] versus female patient/female physician 7·034 [7·146] days). Sagy et al.[^46^](https://paperpile.com/c/3sOdUZ/GAjUR) (N=831) reported no significant association between LOS and physician-patient sex concordance among critically ill patients presenting to the emergency department.

Wallis et al.[^42^](https://paperpile.com/c/3sOdUZ/LFqWH) (n=1,165,711), reported no significant difference in adverse postoperative outcomes between patients managed by sex concordant (i.e. same-sex anesthesiologist and surgeon) versus discordant (i.e. different sex anesthesiologist and surgeon) teams (aOR 1·00; 95% CI: 0·97 to 1·03) among patients requiring common surgical procedures in Canada. Similarly, Becker et al.[^45^](https://paperpile.com/c/3sOdUZ/bzZiv) reported no significant difference in guideline adherence or patient mortality when comparing all-male or mixed-gender care teams. However, none of the included patients were managed by an all-female team. On the other hand, Etherington et al.[^41^](https://paperpile.com/c/3sOdUZ/y9HcM) (n=541,209) studied patients undergoing non-cardiac surgery in Canada and found that physician sex discordance was associated with a lower rate of 1-year mortality (adjusted hazard ratio [aHR] 0·95; 95% CI: 0·91 to 0·99). Specifically, those managed by female surgeons and male anesthesiologists had decreased 1-year mortality versus those treated by all-male teams (aHR 0·90; 95% CI: 0·81 to 0·99).
